# New Insights into the Mechanisms of Embryonic Stem Cell Self-Renewal under Hypoxia: A Multifactorial Analysis Approach

**DOI:** 10.1371/journal.pone.0038963

**Published:** 2012-06-11

**Authors:** Hélder S. C. Barbosa, Tiago G. Fernandes, Tiago P. Dias, Maria Margarida Diogo, Joaquim M. S. Cabral

**Affiliations:** Department of Bioengineering, and Institute for Biotechnology and Bioengineering, Centre for Biological and Chemical Engineering, Instituto Superior Técnico, Technical University of Lisbon, Lisboa, Portugal; Baylor College of Medicine, United States of America

## Abstract

Previous reports have shown that culturing mouse embryonic stem (mES) cells at different oxygen tensions originated different cell proliferation patterns and commitment stages depending on which signaling pathways are activated or inhibited to support the pluripotency state. Herein we provide new insights into the mechanisms by which oxygen is influencing mES cell self-renewal and pluripotency. A multifactorial approach was developed to rationally evaluate the singular and interactive control of MEK/ERK pathway, GSK-3 inhibition, and LIF/STAT3 signaling at physiological and non-physiological oxygen tensions. Collectively, our methodology revealed a significant role of GSK-3-mediated signaling towards maintenance of mES cell pluripotency at lower O_2_ tensions. Given the central role of this signaling pathway, future studies will need to focus on the downstream mechanisms involved in ES cell self-renewal under such conditions, and ultimately how these findings impact human models of pluripotency.

## Introduction

Embryonic stem (ES) cells are pluripotent cells found in the inner cell mass of the blastocyst that are capable of differentiation into the three embryonic germ-layer tissues [1]. This attribute makes ES cells particularly promising in potential applications for the treatment of a variety of diseases [2], [3]. However, to realize this potential a good understanding of the molecular mechanisms that allow efficient ES self-renewal and differentiation is needed.

Mouse ES (mES) cells in particular can be maintained *in vitro* in feeder-free culture conditions with no signs of early lineage commitment via the activation of two distinct signaling pathways: the signal transducer and activator of transcription 3 (STAT3) signaling by the leukemia inhibitory factor (LIF); and via Smad pathway activation [4]. Alternatively, undifferentiated mES cells can also self-renew via the suppression of autocrine fibroblast growth factor-4 (FGF4) signaling that induces mES cell differentiation [5], [6]. This suppression can be accomplished via the inhibition of the mitogen-activated protein kinase (MAPK, MEK/ERK) signaling pathway using small molecule inhibitors such as PD0325901 (PD, PD032) [7]. This inhibition can restrain mES cells from differentiating, although, it is inefficient to support mES cells at clonal densities. To restore this capacity, another kinase inhibitor - CHIR99021 (CHIR), a potent and selective inhibitor of the glycogen synthase kinase-3β (GSK-3β) - needs to be supplemented to the culture medium [7]. In fact, CHIR also restores mES cell viability when excess of MEK/ERK blockade is promoting cell death [7]. However, the solo blockage of GSK-3β renders cells to non-neural differentiation, thus, for efficient self-renewal at low cell densities MEK/ERK signaling pathway inhibitors [7], [8] or LIF [9] are required. GSK-3β itself has many biological functions, yet its inhibition is well known to constitutively stimulate the canonical Wnt/β-Catenin pathway [10].

Regardless of which signaling pathway is initiated or inhibited, the roles of the above mechanisms were uncovered using non-physiological oxygen levels, typically at ∼20% O_2_. Yet, the physiological oxygen tensions to which cells are subjected in the early embryo are much lower [11], [12], making oxygen tension a potentially important factor in the modulation of cell pluripotency and differentiation. Previous work has demonstrated strong links between O_2_ availability, cell proliferation and pluripotency patterns depending on which signaling pathways are activated/inhibited to support mES cell self-renewal. For example, under reduced oxygen levels (1–2% O_2_), STAT3 signaling is down regulated, resulting in a negative effect towards mES cell pluripotency and self-renewal [13], [14]. This is partly due to the hypoxia inducible factor-1α (HIF-1α)-mediated suppression of the LIF-specific receptor (LIFR), attenuating STAT3 phosphorylation, critical for the signaling pathway transduction [13]. Nevertheless, hypoxia did not induce cell differentiation or affected cell proliferation when cells were maintained in feeder-free culture without LIF but with FGF4/ERK and GSK-3β signaling inhibition [15]. More recently, new evidences demonstrated that HIF-1α is also responsible for Wnt/β-catenin signaling modulation at low oxygen levels by enhancing β-catenin activation and expression of downstream effectors (LEF-1 and TCF-1) in non-differentiated cells [16].

**Figure 1:**
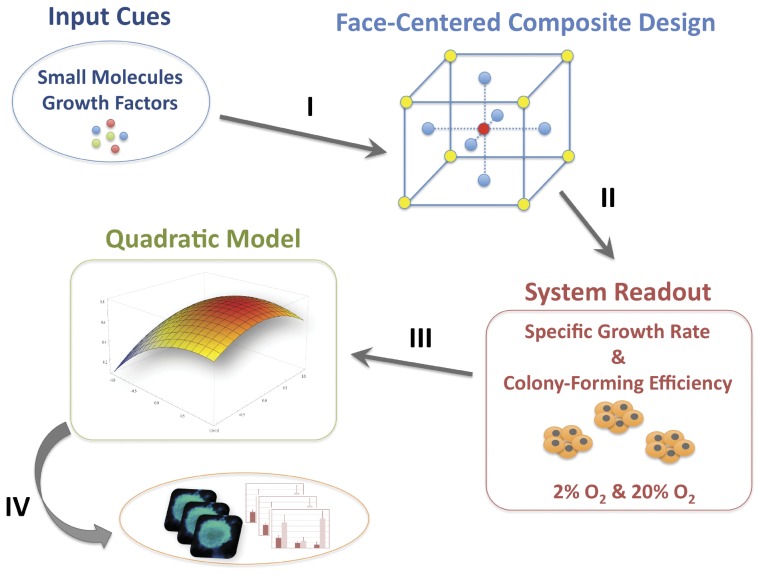
Schematic illustration of the experimental design. Through literature search, two small molecule inhibitors and a cytokine targeting key pathways involved in mES cell pluripotency were selected. Combinations and concentrations of the three signaling modulators were organized using a face-centered composite design (I). Experimental readouts (specific growth rate and colony-forming efficiency) were used to obtain models linking the cellular readouts and the cocktail combinations (II and III). Finally, the models relating small molecule/cytokine-based modulation of signaling pathways and cellular readouts were validated to verify the accuracy of the predicted responses (IV).

These findings indicate that mES cell self-renewal and pluripotency, which are dependent on multifactorial signaling networks, can be differentially influenced by distinct O_2_ levels. To elucidate and dissect how each signaling pathway is influenced at physiological and non-physiological oxygen tensions, we have used a multifactorial approach and response surface methodology (Figure 1). This series of multifactorial experiments enabled the statistical comparison of the concentration-dependent influence of each small molecule/cytokine used for modulating the inhibition of autocrine MEK/ERK pathway (PD), activation of Wnt/β-Catenin pathway through the inhibition of GSK-3β (CHIR), and activation of STAT3 signaling (LIF), in order to determine the synergistic and sole effects of each signaling pathway at different oxygen levels. GSK-3-mediated signaling, in particular, has shown a significant role towards maintenance of mES cells at low oxygen tensions. These results together with reports highlighting the role of Wnt/β-Catenin signaling in human ES cells [17] increase the possibility of translating these findings to human models of pluripotency.

## Results

### Experimental Design

A face-centered composite design (FC-CD) experiment was performed to evaluate the influence of the inhibition of autocrine MEK/ERK pathway (by PD), the activation of Wnt/β-Catenin pathway (by CHIR) and the activation of STAT3 signaling pathway (by LIF) on mES cell expansion and colony-forming capacity at different oxygen tensions (Table S1). In this factorial design, high and low concentration values were selected for each independent variable (PD, CHIR, and LIF concentrations). The selected values were based on the literature [4], [7], where these factors were used individually or in combination in mES cell propagation experiments. We used serum-free medium and focused on longer periods under hypoxia in order to allow cells to fully adapt to the different culture conditions under study.

Cells were then cultured for five consecutive passages at 2 and 20% O_2_ in KO-DMEM/SR as basal medium supplemented with different combinations of the three factors. In parallel, the levels of dissolved O_2_ were measured as a function of time, and the oxygen partial pressure to which cells are exposed (pO_2_ cell) was estimated based on literature values [14] (Figure S1 and File S1). At the end of day 10, the specific growth rate (SGR) was calculated (Methods). The colony-forming efficiency (CFE) was estimated for each condition after culturing the cells during the following six days at low cell densities (200 cells/cm^2^). Table S2 presents specific growth rate values and colony-forming efficiencies for each tested condition.

The mathematical relations of the dependant variables (SGR or CFE) and the three independent variables were modeled using equation 1 (Methods) and the effects with less than 95% of significance (p-value >0.05) were pooled into the residual error term for both oxygen levels. The models were further validated by testing the significance of regression (SOR) and lack of fit (LOF) by analysis of variance (ANOVA) using the Fisher’s statistical test [18]. A F-value greater than F-critical with a p-value lower than 0.05 for SOR, and a F-value lower than F-critical and p-value greater than 0.05 for LOF are an indication of statistical significance at a 95% confidence level. A summary of the ANOVA results obtained for each model is shown in Tables S3 and S4.

### Cell Expansion at 2 and 20% O_2_ Levels

**Figure 2:**
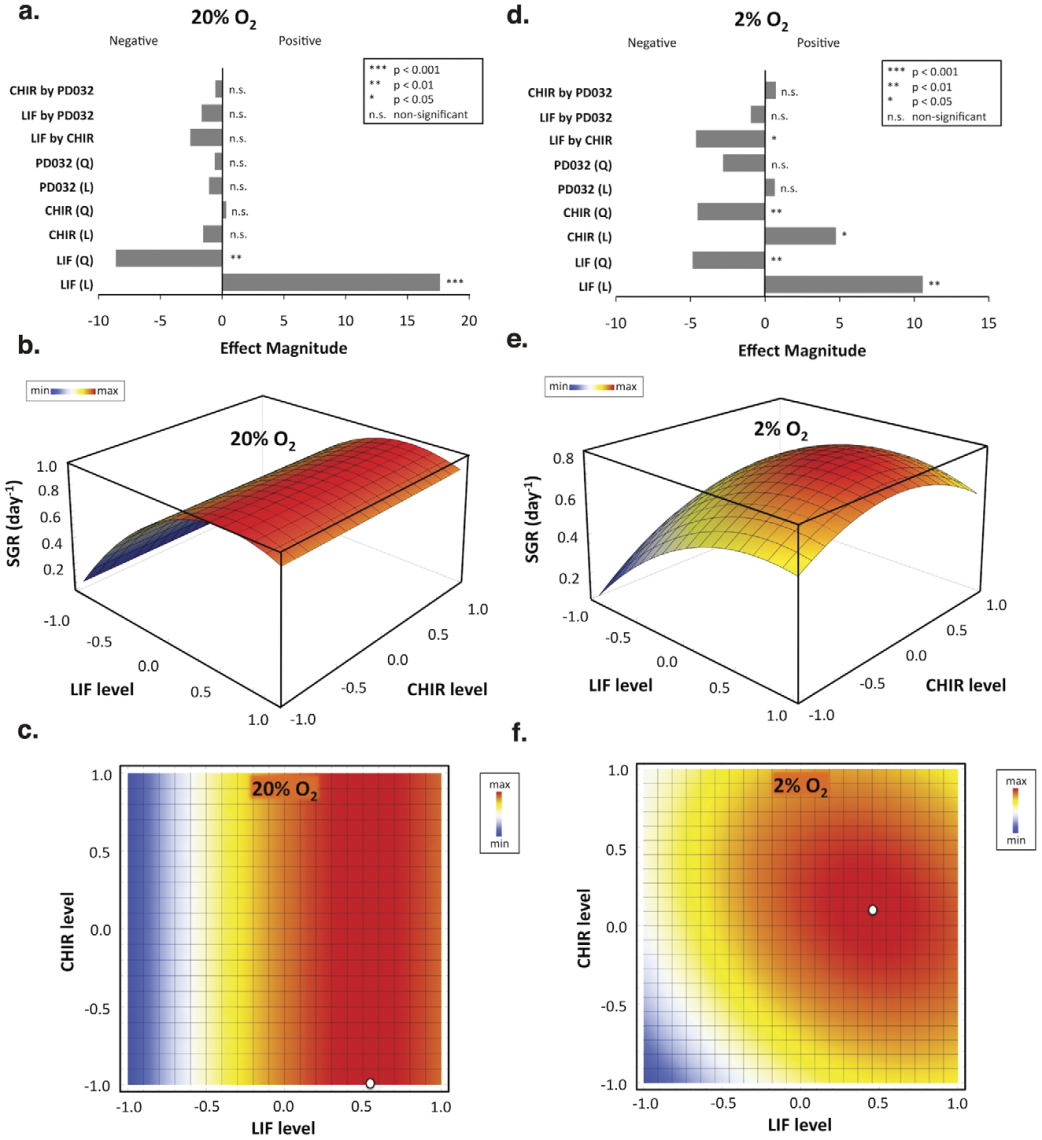
Factorial design models relating signaling input and specific growth rate values of mES cells cultured at different oxygen tensions. mES cells were expanded for a total of 10 days in culture (five consecutive passages) under normoxic (a, b, and c) or hypoxic conditions (d, e, and f). Effect magnitudes of all main factors, second order effects, and two-way interactions were obtained for 20% O_2_ (a) and 2% O_2_ (d). The coefficients of the models were then determined following a sequential backward elimination procedure, where the least significant terms (p>0.05) were eliminated and absorbed into the error. 3D representations for both 20% O_2_ and 2% O_2_ models are shown in (b) and (e), respectively, as a function of LIF and CHIR levels. Two-factor interaction heat maps are also shown for 20% O_2_ (c) and 2% O_2_ (f). White dots represent conditions that maximize the response. For LIF: 0 U/mL (−1 level) ≤ [LIF] ≤1000 U/mL (1 level); for CHIR: 0 µM (−1 level) ≤ [CHIR] ≤6 µM (1 level).

The effects of three independent variables LIF, CHIR and PD on SGR of mES cells were determined using a FC-CD approach. SGR values were fit into a least-squares polynomial regression to estimate the impact of single or combined effects of molecules on mES cell growth. Results from the two-level factorial design indicated that at 20% O_2_ LIF had a significant (p<0.001) and positive effect on SGR (Figure 2a). The second order term of LIF was also significant (p<0.01), and in this case negative indicating a downward concavity of the model. This suggests the existence of a maximum value in the concentration range used in this study. On the other hand, at 2% O_2_ both LIF (p<0.01) and CHIR (p<0.05) had a significant and positive effect on SGR (Figure 2d). Moreover, the second order terms were also significant (p<0.01 for both LIF^2^ and CHIR^2^) and negatives, which again indicates the existence of maximum responses within the concentration range used for these molecules. Interestingly, the combined action of both input signals leads to significant effects on SGR at 2% O_2_ (p<0.05 for LIF by CHIR).

The equations that describe the reduced quadratic models obtained for the SGR response are shown below:

For 20% O_2_ tension,
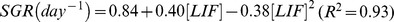
and for 2% O_2_,



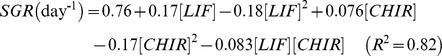
The second order polynomials generated for each oxygen tension described a significant percentage of the experimental data (R^2^ = 0.93 and 0.82 for 20% and 2% O_2_, respectively) with no lack of fit associated (Table S3). Based on each regression models, response surface plots were made for each oxygen level as shown in Figures 2b and 2e. According to the models described above, it is possible to observe that generally mES cells significantly reduce its propagation in serum-free medium when cultured at physiological oxygen levels. Taking the highest SGR at both oxygen levels, this reduction represents a 6.7-times lower yield in total cell number at the end of the tenth day of expansion. This higher mES cell proliferation rate in normoxia was obtained when the culture medium was supplemented with LIF (Figures 2b and 2c). SGR maximum value was reached when LIF was supplemented at ∼760 U/mL (Figure 2c, white dot). Above or below this level, mES cells decrease proliferation in KO-DMEM/SR medium (Figure S2). None of the two small molecule inhibitors (PD and CHIR) at the varied concentrations tested had a significant impact on mES cell expansion at this O_2_ tension, indicating that STAT3 signaling was dominant over MEK/ERK and Wnt/β-Catenin signaling pathways under these conditions.

On the contrary, under hypoxia (Figures 2e and 2f), activation of Wnt/β-Catenin signaling via inhibition of GSK-3 had a significant influence over mES cell expansion. The second order terms of LIF and CHIR in the model were negative suggesting the existence of a maximum value for SGR. Biologically, this indicates an inhibition of mES cell growth when large amounts of LIF and/or CHIR are added to the culture, symptomatic of a possible overload of molecular signals (Figure S2). In hypoxia, culture conditions that maximize mES cell SGR are reached when LIF is supplemented at ∼720 U/mL and CHIR at ∼3 µM (Figure 2f, white dot).

**Figure 3:**
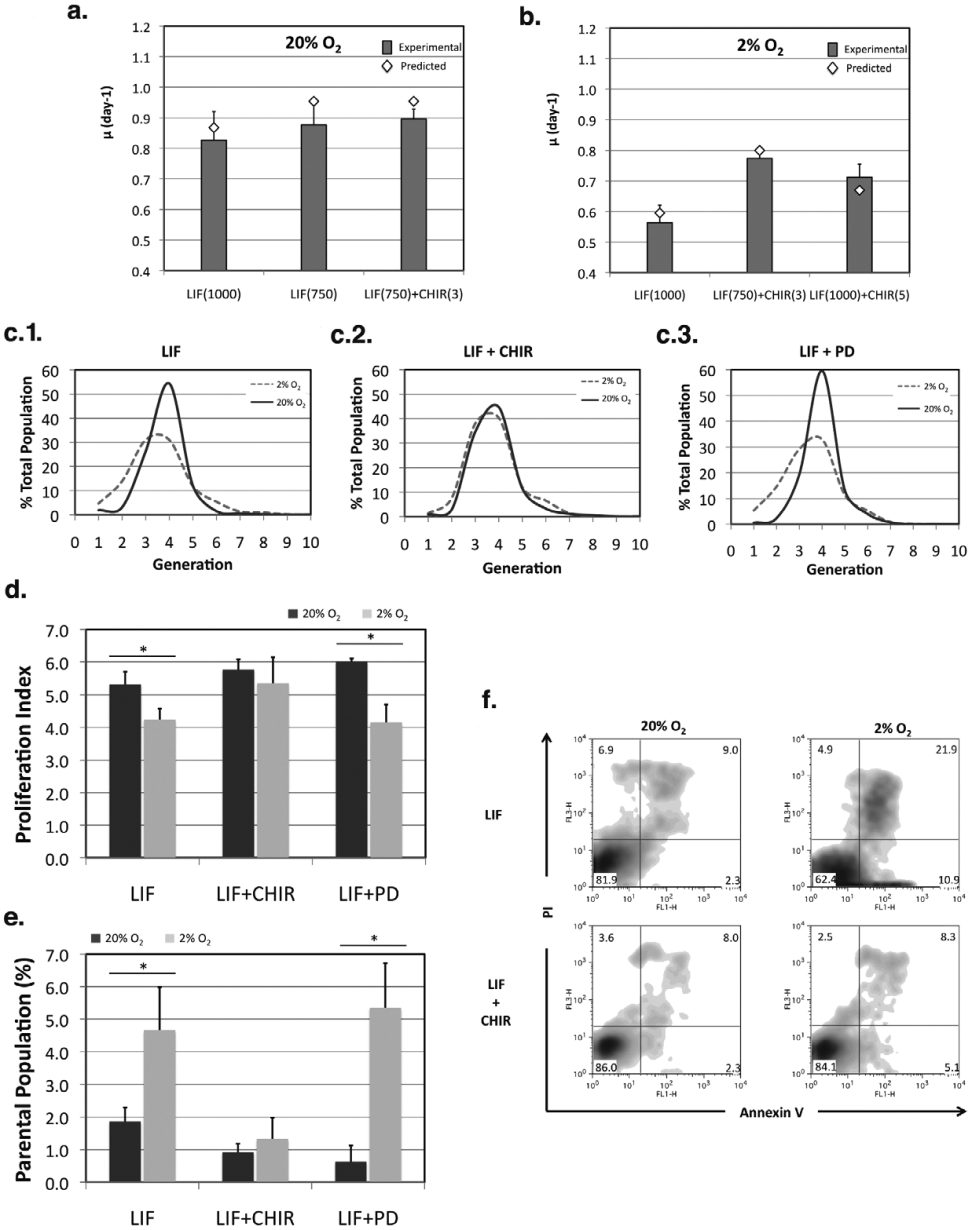
Validation of the model predictions for mES cell proliferation at 20% and 2% O_2_. Specific growth rate values obtained for selected conditions at 20% O_2_ (a) and 2% O_2_ (b). LIF(1000) –1000 U/mL of LIF; LIF(750) –750 U/mL of LIF; LIF(750) + CHIR(3) –750 U/mL of LIF and 3 µM of CHIR; LIF(1000) + CHIR(5) –1000 U/mL of LIF and 5 µM of CHIR (n = 4). Proliferation results were further confirmed by cell division analysis using a PKH fluorescent dye (c). Comparison of the number of cell divisions was made for 20% and 2% O_2_ conditions with LIF-only supplementation (at 1000 U/mL, c.1), LIF + CHIR supplementation (at 1000 U/mL of LIF and 3 µM of CHIR, c.2), and LIF + PD supplementation (at 1000 U/mL of LIF and 0.4 µM of PD, c.3). The proliferation index (d) and percentage of parental population (e) were also calculated using ModFit software, and further comparison was made for the same culture conditions at 20% and 2% of O_2_ (n = 3). (f) Cellular apoptosis and necrosis was further evaluated using flow cytometry following FITC-Annexin V/Propidium Iodide staining for LIF and LIF + CHIR conditions at 20% and 2% O_2_. Results are presented as mean ± SEM. * p<0.05.

Confirmatory experiments were conducted to validate the predicted response curves obtained from the models. mES cells were expanded at 20% O_2_ using KO-DMEM/SR medium supplemented with 1000 U/mL of LIF, 750 U/mL of LIF, or 750 U/mL of LIF and 3 µM of CHIR. These conditions were selected because 1000 U/mL of LIF are typically used for routine expansion of these cells. However, our model predicted a maximization of cell expansion at 750 U/mL of LIF, and CHIR supplementation was performed at 3 µM to evaluate if in fact LIF was the only significant input factor at 20% O_2_. The validation experiments confirmed the predictions of the model, including the increase in SGR at 750 U/mL of LIF and no significant effect of CHIR supplementation under normoxia (Figure 3a). For validation of the predicted responses at 2% O_2_ an additional condition at 1000 U/mL of LIF and 5 µM of CHIR was also used to represent suboptimal conditions for cell expansion under hypoxia. The predictions of the model were also confirmed, particularly the need for simultaneous stimulation of STAT3 signaling and inhibition of GSK-3 for optimal mES cell expansion at reduced oxygen tension (Figure 3b). LIF supplementation alone caused reduction of SGR, which was recovered when both LIF and CHIR were present in the culture medium at 750 U/mL and 3 µM, respectively (conditions close to the predicted maximum at 720 U/mL of LIF and 3 µM CHIR). Further increase in LIF and CHIR input resulted in a reduction of SGR, as also predicted by the model.

All proliferation results were further confirmed by cell division analysis using a PKH fluorescent dye [19]. The determination of the number of cell divisions during the last passage in culture by flow cytometry revealed that cells expanded at low O_2_ levels divided fewer times when LIF alone was present in the culture medium (Figure 3c.1). Similar results were obtained when PD was also added in culture (Figure 3c.3). However, supplementation with the GSK-3 inhibitor CHIR in addition to LIF resulted in similar percentages of cells in later generations as compared with 20% O_2_ using the same medium supplementation (Figure 3c.2). Furthermore, comparison of the proliferation index reveals a statistically significant reduction of this parameter at 2% O_2_ when LIF alone (p = 0.032), or in combination with PD (p = 0.029), is used to maintain mES cells (Figure 3d). This reduction is not observed when LIF and CHIR are both supplemented to the culture. Analysis of the fraction of cells that did not divide (*i.e.* the parental population) also revealed a significant proportion (p = 0.030) of non-proliferating cells at 2% O_2_ in culture conditions supplemented with LIF, or LIF and PD (Figure 3e). Our proliferation analysis thus provides evidence that addition of CHIR caused an increase in cell divisions at low O_2_ levels as compared to LIF-only conditions under the same oxygen tension. Furthermore, low oxygen tension resulted in a decrease in cell viability throughout time in culture when LIF alone was used to support mES cell expansion. This reduction in viability under hypoxia was proportional to an increase in the fraction of apoptotic and necrotic cells (Figure 3f). When CHIR was supplemented to the medium, however, no differences could be observed between cells expanded at 20% and 2% O_2_. Similar observations were made when cells were maintained with LIF and PD or with the two chemical inhibitors and LIF (2i+LIF) (Figure S3). Together with a decrease in the number of cell divisions, the reduction in cell viability contributes to reduced cell yields and lower SGRs obtained at low oxygen levels in conditions supplemented with LIF alone.

According to reports in the literature, at low oxygen levels STAT3 signaling is partially downregulated, due to the activation of HIF-1 that binds to the LIFR promoter leading to a suppression of LIFR transcription [13]. Under these conditions, the inhibition of GSK-3β and consequent activation of Wnt/β-Catenin pathway has been shown to contribute to mES cell self-renewal [7], [17]. Here, the sole presence of LIF resulted in very low proliferation rates under hypoxia. However, as predicted by the factorial design models, we confirmed that STAT3 activation was necessary to reach maximum expansion in hypoxia, with no need for inhibiting the MEK/ERK signaling pathway. Overall, these findings indicate that Wnt signaling mediated by the canonical pathway is not absolutely sufficient and requires a synergistic action with LIF, but not MEK/ERK inhibition, to reach maximum proliferation of mouse ES cells under low oxygen levels.

### Colony-forming Efficiency (CFE) at 2 and 20% O_2_ Levels

We next evaluated the capacity of different culture conditions to support mES growth at clonal densities, as a measure of the capacity of those conditions to promote cell self-renewal. Therefore, high efficiencies of colony formation indicate that mES cells have a high percentage of survival and can generate a high number of colonies, suggesting that the culture conditions employed can better support mES cell self-renewal.

**Figure 4:**
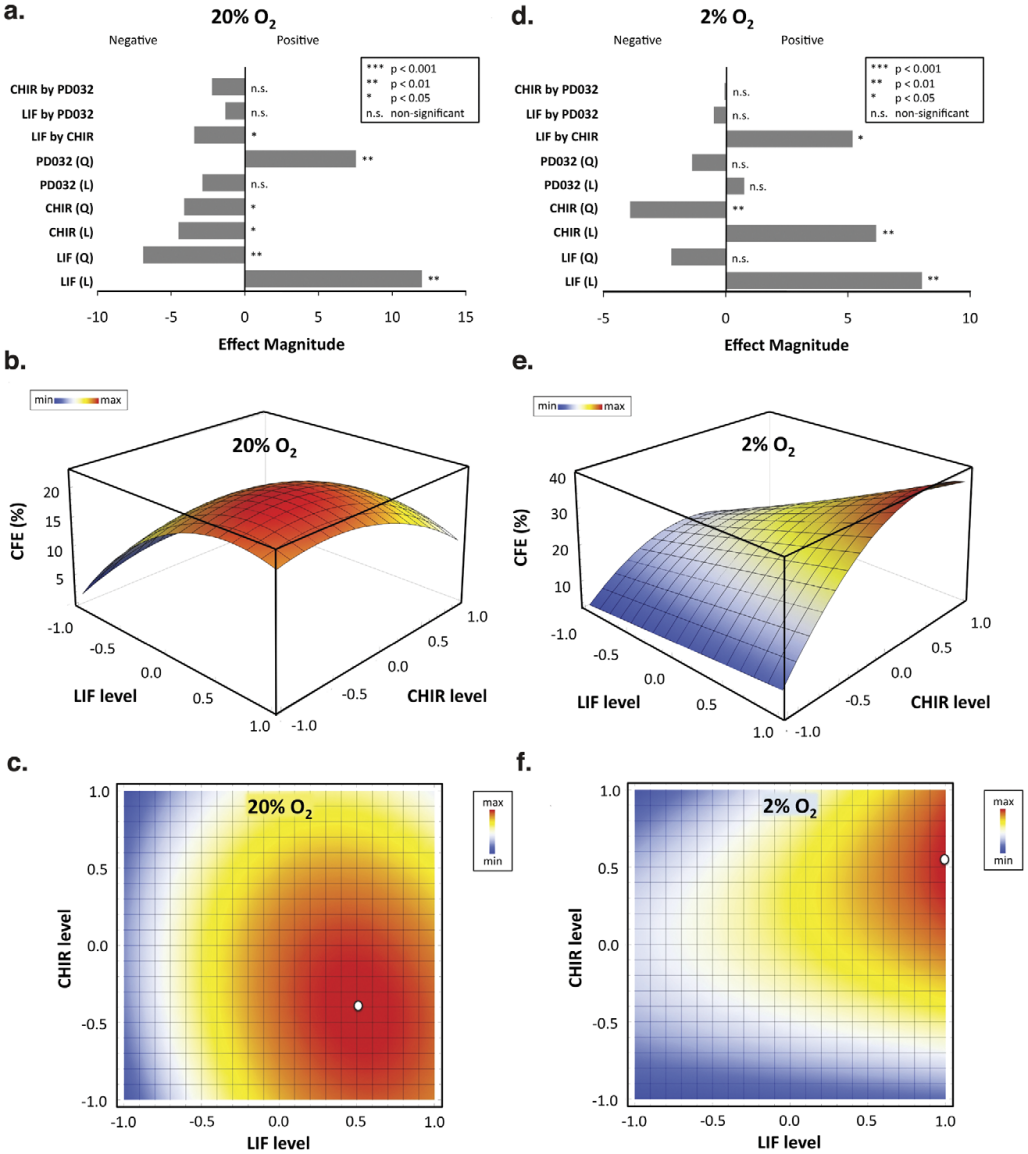
Factorial design models relating signaling input and colony-forming efficiency of mES cells cultured at different oxygen tensions. After cell expansion for 10 days, mES cells were plated using the same conditions at clonal densities under normoxia (a, b, and c) or hypoxia (d, e, and f). Effect magnitudes of all main factors, second order effects, and two-way interactions were obtained for 20% O_2_ (a) and 2% O_2_ (d). The coefficients of the models were then determined following a sequential backward elimination procedure, where the least significant terms (p>0.05) were eliminated and absorbed into the error. 3D representations for both 20% O_2_ and 2% O_2_ models are shown in (b) and (e), respectively, as a function of LIF and CHIR levels ([PD] = 0 µM). Two-factor interaction heat maps are also shown for 20% O_2_ (c) and 2% O_2_ (f). White dots represent conditions that maximize the response. For LIF: 0 U/mL (−1 level) ≤ [LIF] ≤1000 U/mL (1 level); for CHIR: 0 µM (−1 level) ≤ [CHIR] ≤6 µM (1 level); for PD: [PD] = 0 µM (−1 level).

CFE values were fit into a least-squares polynomial regression to estimate the impact of single or combined effects of the molecules. Results from the two-level factorial design indicated that at 20% O_2_ both LIF and CHIR main effects were significant (p<0.01 and p<0.05 for LIF and CHIR, respectively). Similarly, both second order terms were also significant (p<0.01 for LIF^2^ and p<0.05 for CHIR^2^) and negative (Figure 4a), pointing to the existence of a maximum value in the concentration range used for both input variables. Moreover, the synergistic activity of LIF and CHIR leads to significant effects on CFE at 20% O_2_ (LIF by CHIR, p<0.05). In addition, it is also possible to observe that PD main effect was not significant for CFE response under normoxia. Nevertheless, since the quadratic term (PD^2^) was considered significant (p<0.01), the main effect of this input variable was reintroduced in the model with no influence in the LOF test that remained non-significant (Table S4). For 2% O_2_ both LIF (p<0.01) and CHIR (p<0.01) had a significant and positive effect on CFE (Figure 4d). Moreover, the second order term of CHIR was also significant (p<0.01) and negative, which again indicates the existence of a maximum CFE response. Since LIF had a positive main effect, in the absence of a significant second order term (p = 0.113), CFE response to this input factor was linear reaching the maximum value at the highest concentration tested (1000 U/mL) (Figure S4). The combined action of LIF and CHIR also leads to significant effects on CFE at 2% O_2_ (LIF by CHIR, p<0.05).

The equations that describe the reduced quadratic models obtained for the CFE response are shown below:

For 20% O_2_ tension.
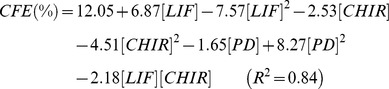
and for 2% O_2_



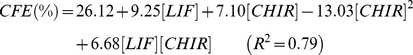
The R^2^ values indicate a good fit between the model and the experimental results (R^2^ = 0.84 and 0.79 for 20% and 2% O_2_, respectively). The slightly lower R^2^ value for CFE in hypoxia is still indicating a good adjustment, particularly when taking into account the common culture variability linked to biological samples. Nevertheless, both models were validated using the lack of fit (LOF) test (Table S4). Thus, we can be confident that the model is explaining the variability in the data. Based on each regression model, response surface plots were made for each oxygen level (Figures 4b and 4e). Culturing mES cells at low density in normoxia resulted that all independent variables had a significant influence on the cloning forming efficiency. Particularly, the model indicates a synergistic effect through the activation of LIF/STAT3 and Wnt/β-Catenin signaling pathways. At normoxia, relatively high colony formation efficiencies (∼25%) were obtained when using intermediate concentrations of CHIR (2–3 µM) in conjunction with LIF at ∼750 U/mL (Figure S4). Similar results were achieved when MEK/ERK signaling inhibitor was supplement at higher concentrations (PD = 0.8 µM) in combination with LIF and lacking CHIR, but with lower CFE values when compared to LIF-only conditions (Figures S4 and S5). These results show that higher colony formation efficiencies are obtained in the presence of LIF alone, or in combination with only one small molecule inhibitor (of GSK-3 or MEK/ERK). CFE is not improved when the two inhibitors are used simultaneously, possibly due to an overload of molecular signaling under these conditions (Figure S4). Consequently, at 20% O_2_, culture conditions that maximize CFE in the absence of PD are reached when LIF is supplemented into culture medium at ∼750 U/mL and CHIR at ∼2 µM (Figure 4c, white dot).

Similar to SGR read-out, input provided by both CHIR and LIF are fundamental to obtain high colony formation efficiencies at low oxygen tensions (Figures 4e and 4f). Biologically, this indicates an absolute requirement for the synergistic action of both LIF/STAT3 and Wnt/β-Catenin signaling (through inhibition of GSK-3) for the maintenance of the pluripotent state at reduced oxygen tensions. Since STAT3 signaling is partially downregulated due to inhibition of LIFR transcription by HIF-1 [13] at low oxygen tensions, GSK-3 inhibition appears to be an absolute requirement for the maintenance of the pluripotent state. Thus, in hypoxia, LIF stimulation is only effective when GSK3 inhibition is engaged, which probably reflects basal activity of STAT3 signaling and synergy with GSK3 inhibition through an unknown mechanism. Therefore, in hypoxia, culture conditions that maximize CFE in mES cells are reached when LIF is supplemented into culture medium at 1000 U/mL and CHIR at ∼5 µM (Figure 4f, white dot, and Figure S4). Interestingly, the models predict that under low oxygen levels a nearly two times higher overall CFE is obtained as compared to normoxic conditions (Table S2). This indicates that physiological oxygen tensions favor mES cell “stemness”, through the capacity to promote mES colony formation at low cell densities, given that appropriate input signaling is used to stabilize the pluripotency state [7], [20].

**Figure 5:**
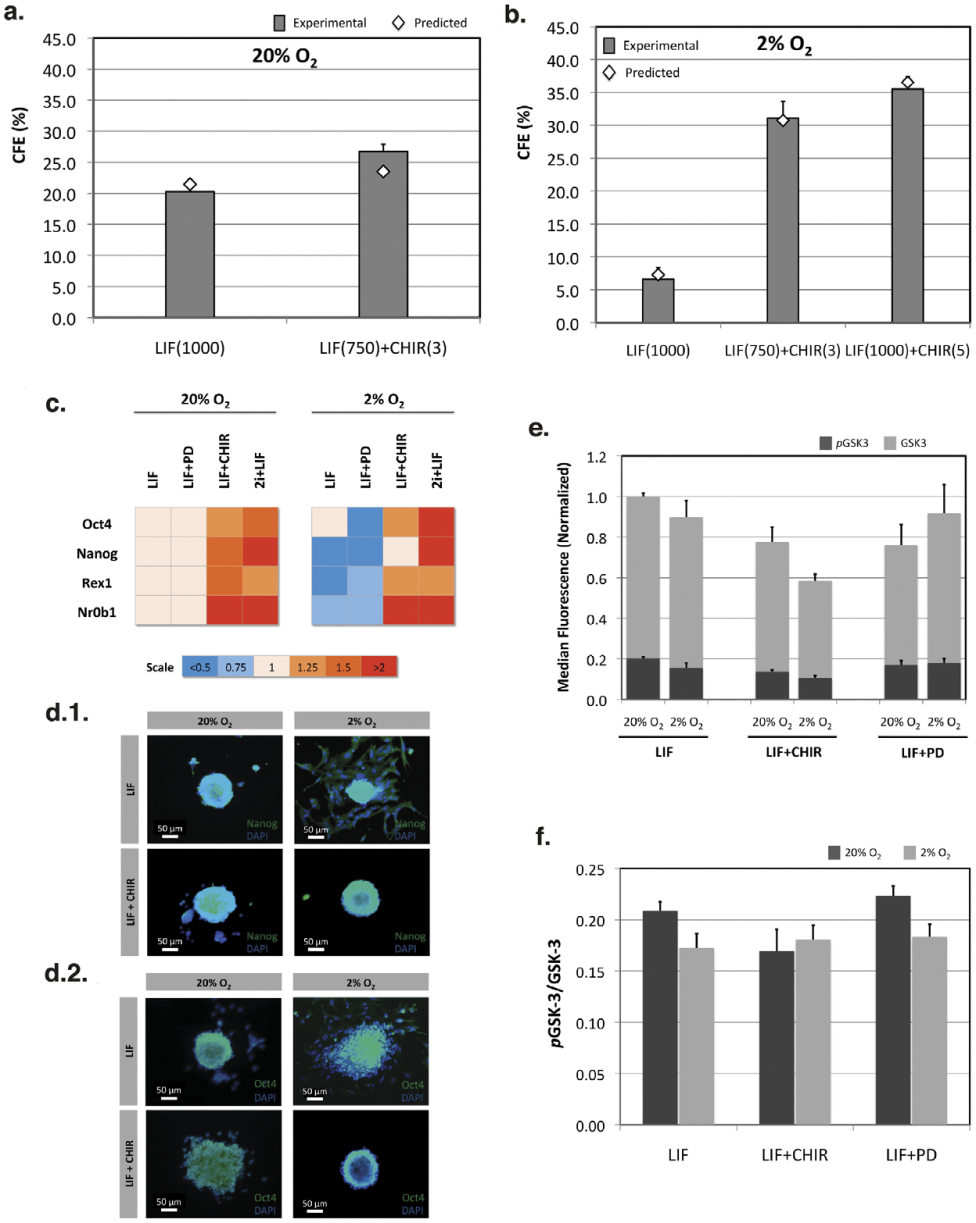
Validation of the model predictions for mES cell colony-forming efficiencies confirm enhanced self-renewal at reduced oxygen levels with GSK-3 inhibition. Colony-forming efficiencies obtained for selected conditions at 20% O_2_ (a) and 2% O_2_ (b). LIF(1000) –1000 U/mL of LIF; LIF(750) + CHIR(3) –750 U/mL of LIF and 3 µM of CHIR; LIF(1000) + CHIR(5) –1000 U/mL of LIF and 5 µM of CHIR (n = 6). Pluripotency markers were also evaluated by quantitative PCR (c) or immunofluorescence staining (d). Heat-maps show relative expression levels of key pluripotency genes (c), and colonies were stained for Nanog (d.1) or Oct4 (d.2) and nuclear marker DAPI (scale bar: 50 µm). The positive effects of GSK-3 inhibition could be correlated with a decrease in total and phosphorylated protein levels at 20% and 2% O_2_ (e). Furthermore, the phospho-to-total GSK-3 ratio at 2% O_2_ was slightly reduced for both LIF and LIF + PD conditions as compared to 20% O_2_, indicating a higher proportion of active GSK-3 (f) (n = 3). Results are presented as mean ± SEM. LIF –1000 U/mL of LIF; LIF + CHIR –1000 U/mL of LIF and 3 µM of CHIR; LIF + PD –1000 U/mL of LIF and 0.4 µM of PD.

Validation experiments were carried out to assess the accuracy of the predicted response curves obtained from our models. The capacity of different culture conditions to support mES cell self-renewal by growing these cells at clonal densities was further evaluated for selected conditions. We again tested conditions at 750 U/mL of LIF and 3 µM of CHIR, and at 1000 U/mL of LIF alone. These conditions are close to the predicted maximization of CFE (∼750 U/mL of LIF and 2 µM CHIR), or the typically used LIF supplementation for routine expansion of these cells (1000 U/mL). Generically, the validation experiments confirmed the predictions of the model (Figure 5a). At 2% O_2_ an additional condition at 1000 U/mL of LIF and 5 µM of CHIR was also used to represent the maximum CFE response under hypoxia. Model predictions were also confirmed in this case, particularly the need for synergistic activity of STAT3 signaling and inhibition of GSK-3 for optimal clonogenic ability at reduced oxygen tension (Figure 5b). LIF supplementation alone caused a marked reduction of CFE, which was recovered when both LIF and CHIR were present in the culture medium at 750 U/mL and 3 µM, respectively. Further increase in LIF and CHIR input resulted in the highest CFE value, as also predicted by the model.

The CFE results were further complemented with molecular and phenotypic analyses in order to better characterize the pluripotent status of mES cells maintained at different oxygen tensions and particular combinations of signaling modulators. Quantitative real-time polymerase chain reaction (RT-PCR) was performed to evaluate the relative expression of pluripotency marker genes (Table S5) after cell expansion at different conditions (Figure 5c and Figure S6). The relative expression of pluripotency markers (*Oct3/4*, *Nanog*, *Rex1* and *Nr0b1*) was reduced under hypoxia when LIF or LIF and PD were supplemented to the culture medium. However, when CHIR is present, the relative expression of these markers is maintained or increased, which confirms the need for GSK-3 inhibition to stabilize the pluripotency state at reduced oxygen levels. At the same time, input provided by both small-molecule inhibitors and LIF (2i + LIF) did not cause a marked increase in the expression of pluripotency markers when compared with LIF + CHIR conditions at 2% O_2_. RT-PCR analysis also shows a positive effect of CHIR together with LIF at 20% O_2_. Globally, the RT-PCR data confirmed the major predictions obtained from our FC-CD models, namely the importance of GSK-3 inhibition to prevent downregulation of key pluripotency genes at reduced oxygen tensions. Interestingly, at 2% O_2_ the absence of CHIR resulted in colonies with differentiated morphology (Figure 5d) as determined by immunocytochemistry analysis. When GSK-3 inhibition is used together with LIF the colonies retain their typical compact morphology with both pluripotency markers localized in the cell nucleus and existing at high levels even in hypoxic conditions. Inhibition of MEK/ERK instead is not able to recover colony integrity at 2% O_2_ (Figure S7), similar to what was observed with LIF-only supplementation.

### GSK-3 Inhibition Enhances Self-renewal of mES Cells at Reduced Oxygen Levels

GSK-3 is a multifunctional kinase that acts as a downstream regulatory switch, determining the output of distinct signaling pathways [21]. GSK-3 is generally active in resting cells and it is inhibited in response to different stimuli [22]. Phosphorylation of GSK-3 at serines-9 and -21 (S9/S21) causes inhibition of GSK-3 activity [23], thus allowing the translocation of its target β-catenin to the nucleus. Therefore, this protein kinase plays a central role in Wnt signaling [24], [25], [26]. According to our results, GSK-3 inhibition is fundamental to maintain both proliferation and self-renewal of mES cells at reduced oxygen tensions. Therefore, we have also analyzed the levels of total GSK-3 and phospho-GSK-3 by flow cytometry for different conditions (Figures 5e and S8). The median fluorescence intensities were obtained for each sample and normalized to the levels of total GSK-3 protein existing at 20% O_2_ in the presence of LIF. When CHIR was present in the culture medium together with LIF, we observed an overall reduction of the total GSK-3 levels either at 20% (p = 0.078) or 2% O_2_ (p = 0.008) as compared to the levels obtained in the presence of LIF at normoxia. The levels of phosphorylated protein were also reduced in these conditions (p = 0.008 for 20% and 2% O_2_) (Figure S8). This result points to a global inhibition of GSK-3 in response to CHIR, and correlates with the positive effects of this inhibition at reduced oxygen levels. CHIR is a highly selective cell-permeable compound that acts as potent ATP-competitor [27], [28], and thus its inhibition mechanism does not depend on phosphorylation of GSK-3. As a result, the ratio of phospho-GSK-3 to total protein remains unchanged at 20% and 2% O_2_ in LIF + CHIR conditions (Figure 5f). On the other hand, while the levels of total GSK-3 remained mostly unchanged when LIF or LIF and PD were supplemented to the culture medium either at normoxia or hypoxia (Figures 5e and S8), it was possible to observe a reduction in the phospho-GSK-3 to total GSK-3 ratio at 2% O_2_ for both input treatments (p = 0.089 for LIF and p = 0.067 for LIF + PD, as compared to each condition at 20% O_2_) (Figure 5f). Despite the slender differences, this points to a higher proportion of active GSK-3 and thus a reduction of downstream signaling cascade, partially explaining the poor performance of mES cells when cultured with these input factors at reduced oxygen tensions.

## Discussion

We have developed a framework based on the rational delineation of signaling input using a multifactorial approach and response surface methodology to evaluate how different signaling pathways are functioning at physiological and non-physiological oxygen tensions (Figure 1). This strategy proved valuable, and similar methodologies could be directed in the future towards measuring more complex biological behaviors. In fact, statistical design of experiments (DOE) is a powerful technique that has been only minimally employed in studying complex biological questions [29], [30], and to our best knowledge no systematic DOE analysis has been developed before to interrogate how different signaling pathways are responding to different environmental input. In the future, it would be essential to devise microscale strategies allowing parallel interrogation of multiple conditions and multiplexed analysis of cellular outcomes to evaluate the effects of different stimuli in pluripotent stem cells [31], [32], [33].

Consistent with previous data [15], our approach predicted the need for synergistic activation of STAT3 signaling and inhibition of GSK-3 for optimal maintenance of pluripotent mES cells at reduced oxygen tension (Figures 2 and 4). In fact, oxygen tension is a critical component of the stem cell niche [34], and cell proliferation/quiescence seems to be regulated by physiological gradients of oxygen [35]. In our system, hypoxia significantly reduced the cumulative fold increase of mES cells (Figure S9), and maximum specific growth rates at 2% O_2_ were only rescued by GSK3 inhibition in the presence of LIF. Interestingly, both LIF/STAT3 and GSK-3 are involved in the regulation of the transcription factor Myc [36], [37], [38]. This factor regulates and coordinates the expression of many genes involved in cell survival and proliferation. Therefore, it is possible that partial recovery of normal proliferation rates and viabilities at 2% O_2_ in the presence of CHIR and LIF (Figure 3) may be due to a Myc-dependent mechanism. However, additional efforts must be developed to further explore this possibility.

GSK-3 inhibition has also proved fundamental in supporting mES cell self-renewal and pluripotency under hypoxia (Figures 4 and 5). Previous reports provided evidence suggesting that hypoxic conditions prompt mES cells for early commitment when LIF alone is used to maintain cell pluripotency [13], [15]. Moreover, involvement of GSK-3 in regulation of murine and human embryonic stem cell self-renewal has been already proposed [17], [39]. Therefore, synergy between STAT3 signaling and Wnt/β-Catenin is fundamental in maintaining self-renewal of mES cells [9], and in our system CHIR supplementation alone is not able to maintain normal cell proliferation and preservation of the pluripotency state (Figures S9 and S10). Nevertheless, the downstream mechanism that regulates mES cell pluripotency at reduced oxygen tensions was not uncovered using our methodology, and additional studies are needed. Recent studies, however, have shown that GSK-3 inhibition results in β-catenin stabilization and mediate the intricate balance between Tcf3-repression and Tcf1-induced control of the pluripotency network [40], [41]. β-catenin stabilization through GSK-3 inhibition may also result in a Tcf-independent mechanism, directly regulating the core components of the pluripotency transcriptional network (*e.g.* Oct4) [42].

Cross talk and synergistic activity of different signaling pathways, such as GSK-3 inhibition and MEK signaling, raise additional levels of complexity. In fact, GSK-3 is a negative regulator of proteins involved in metabolism, transcription, translation, cell cycle, apoptosis and signal transduction, and therefore, due to the central role in many biological responses [22], its inhibition has potentially much broader effects than canonical Wnt signaling. In addition to the described convergence of Wnt/β-catenin and LIF/STAT3 signaling [43], other pathways such as the phosphoinositide 3-kinase (PI3K) pathway may be involved in the regulation of mES cell self-renewal [44]. For example, Ras-dependent PI3K pathway is also critical for controlling Myc protein accumulation, likely through the control of GSK-3 activity [38]. Hypoxia in particular initiates intracellular signaling events leading to the activation of HIFs [45], which appear to modulate Wnt/β-catenin signaling [16] and downregulation of STAT3 signaling [13]. Moreover, in hypoxia, HIF-1α stabilization appears to connect PI3K/Akt pathway and GSK3 [46], further increasing the complexity of signaling events that are involved in the maintenance of the pluripotency state under these conditions. In fact, HIF function is primarily regulated by the α subunit stability, but while HIF-2α promotes hypoxic cell proliferation by enhancing c-myc transcriptional activity [47], [48], HIF-1α antagonizes Myc function [49], highlighting the fact that different HIF proteins differently regulate specific sets of genes. For example, in human ES cells and continuous hypoxia, HIF-1α is transiently expressed with a peak at 48 hours, following a decrease to almost undetectable levels [50]. This suggests that HIF-1α may have a role in the adaptive response to hypoxia, being substituted by other subunits later on. HIF-2α in particular seems to substitute HIF-1α in response to long-term hypoxia, and regulates a different set of genes [51]. HIF-2α-target genes include Oct4, a transcription factor essential for maintaining stem cell pluripotency [52]. Due to this complex signaling landscape, current cellular biology paradigms are clearly not sufficient to unravel such intricate mechanisms, and coherent quantitative understanding of stem cell fate at the systems level is necessary [53].

In conclusion, based on the rational delineation of signaling input using a multifactorial approach and response surface methodology, we were able to statistically evaluate the synergistic and sole effects of LIF/STAT3, Wnt/β-catenin and MEK/ERK signaling pathways at physiological and non-physiological oxygen tensions. This framework provided new information on how oxygen influences mES cell self-renewal and pluripotency, and GSK-3-mediated signaling, in particular, has shown a significant role towards maintenance of mES cells at low oxygen tensions. Given the role of Wnt/β-Catenin signaling in human ES cells [17], future studies need to address how these findings impact human stem cell pluripotency.

## Methods

### Expansion of mES Cells

Upon thawing, mES cells were expanded for three consecutive passages at authentic ground state on gelatinized tissue culture plates (0.1% (v/v) gelatin in phosphate buffered saline (PBS; Gibco), diluted from a 2% stock solution; Sigma) using iSTEM serum-free medium (Stem Cell Sciences Inc.). This culture medium is supplemented with three chemical inhibitors that eliminate differentiation signals, sustaining mouse ES cell self-renewal and maintaining the pure ES cell ground-state, as previously described [7]. mES cells were then transferred to Knockout Dulbecco’s modified Eagle’s medium (DMEM; Gibco) supplemented with 15% (v/v) Knockout serum-replacement (SR; according to the manufacturer’s instructions, Gibco), 2 mM glutamine (Gibco), 1% (v/v) penicillin (50 U/mL)/streptomycin (50 µg/mL) (Pen/Strep; Gibco), 1% (v/v) nonessential amino acids (Sigma), and 0.1 mM β-mercaptoethanol (β-ME; Sigma). Knockout serum-free medium (KO-DMEM/SR) was supplemented with LIF (Millipore), CHIR99021 and PD0325901 (Stemgent) according to the two-level face-centered cube design (FC-CD) experiment described in Table S1.

The cells were then cultured for five consecutive passages (10 days) at different oxygen tensions as previously described [15]. Briefly, cells were cultured under atmospheric oxygen tension (20% O_2_) or reduced oxygen atmosphere (hypoxic chamber connected to a Proox Model 21 controller set up at 2% O_2_; BioSpherix) at 37°C in a 5% CO_2_ humidified incubator. Every 2 days, cells were dissociated using Accutase solution (Sigma) and re-plated at the same initial cell density (25 000 cells/cm^2^). At each passage, viable and dead cell numbers were determined by counting in a hemocytometer under an optical microscope using the trypan blue dye exclusion test (Gibco). Assuming exponential growth, the apparent specific growth rate, μ (day^−1^), was calculated as 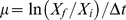, where X_f_ and X_i_ are the viable cell numbers at the beginning and at the end of any given time interval (Δt). The fold increase in total cell number was defined as the ratio between final and initial viable cell numbers for each passage. The cumulative fold increase was calculated as the product of fold increase values obtained at the end of the fifth passage.

Additional information regarding the cell line used in experiments, cell division assay, apoptosis assay, intracellular staining for immunofluorescence microscopy and flow cytometry, quantitative RT-PCR, and determination of dissolved oxygen levels in liquid medium and pO_2_ cell can be found in File S1 section.

### Pluripotent Stem Cell Expansion at Clonal Densities

After the fifth passage cells were seeded under clonal density, 200 cells/cm^2^, on gelatinized 24-well tissue culture plates and cultured 6 days in the same culture conditions as described in Table S1, at either 20% and 2% O_2_ tensions. Medium was replaced every two days. In the case of incubation at 2% O_2_, culture plates were removed from hypoxic chamber and medium changes were made at normal oxygen tension. The time-dependent response of the liquid-phase oxygen levels in tissue culture plates is shown in Figure S1.A. Once the culture plates were transferred to the hypoxic chamber, it was possible to observe a swift reduction in dissolved oxygen within the first hour. The dissolved O_2_ continued to drop at later times, and eventually reached equilibrium with the gas phase after approximately 5 hours of incubation. Therefore, in our system, cells cultured under hypoxia are exposed to higher O_2_ levels for at least 5 h once every 2 days. This is a limitation of the system and effects on cellular responses due to this procedure cannot be excluded. On day 6 the number of colonies formed were counted and the efficiency in colony formation was determined as the ratio between the number of colonies formed and the number of cells seeded on each well/condition (400 cells).

### Experimental Design

The effects of three independent variables, LIF, CHIR99021 and PD0325901, on the specific growth rate and the efficiency in colony formation of mES were determined using a face-centered composite design (FC-CD) approach using STATISTICA software (StatSoft, Tulsa, OK). Each independent variable was evaluated at three different coded levels (low (−1), central (0) and high (+1)) as portrayed in Table S1 and combined in a FC-CD design set up described as: 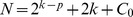, where N is the number of experiments, k is the number of variables (k = 3), p the fractionalization number (in a full design, p = 0) and C_0_ is the number of central points, that provides estimation of the experimental error. Accordingly, a total of 18 [2^3−0^+ (2×3) +4] independent experiments were performed for each oxygen tension tested.

### Statistical Analysis

The data was fitted to a full quadratic model (including linear and non-linear effects, plus two-way interactions) as follow:
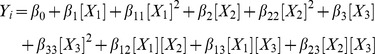
where Y*_i_* is the response measured or dependent variable (specific growth rate and colony-forming efficiency), β*_0_* is the intersect; β*_1_*, β*_2_* and β*_3_* are the linear main effects, β*_11_*, β*_22_* and β*_33_* are the quadratic coefficients and β*_12_*, β*_13_* and β*_23_* are the coefficients for the second order interactions. The statistical significance of each model was evaluated by ANOVA using the Fisher’s statistical test. Regression coefficients were determined following a sequential backward elimination procedure, where the least significant terms, less than 95% significance (p>0.05), in each step were eliminated and absorbed into the error.

Validation results are presented as mean ± standard error of mean (SEM). Comparisons between experimental results were determined by Mann-Whitney test for independent samples, when appropriate. A p-value less than 0.05 was considered statistically significant.

## Supporting Information

Figure S1 **Determination of dissolved oxygen levels in liquid culture medium and estimation of oxygen partial pressure at the cell level (pO_2_ cell).** Measurements were made in 1 mL of KO-DMEM/SR medium and O_2_ levels were plotted as a function of time (A). The liquid was initially equilibrated at atmospheric oxygen levels, and the plate was then transferred to a humidified incubator at different levels of oxygen in the gaseous phase. Under 20% O_2_ the dissolved oxygen at the bottom of the culture medium layer was similar to the gas phase tension. As oxygen gas phase levels were reduced (to 2% O_2_) it was possible to observe a swift reduction in dissolved oxygen within the first hour. The dissolved O_2_ continued to drop at later times, and eventually reached equilibrium with the gas phase after approximately 5 hours of incubation. Based on the literature, pO_2_ cell values were obtained for low cell densities (1×10^4^ cells/cm^2^) and high cell densities (2×10^5^ cells/cm^2^) and plotted as a function of oxygen levels in the gas phase (B). The corresponding response curves are represented in the graphic for both cell densities. The grey area between both curves represents the expected decrease in pO_2_ cell levels with increasing cell densities at lower oxygen tensions (up to 5% O_2_). The grey arrow represents the estimated pO_2_ cell range for our system at 2% O_2_.
(TIF)

Figure S2 **Maximum specific growth rate profiles obtained for 20% and 2% O_2_ FC-CD models.** Each graphic represents the predicted variation of the maximum SGR obtained by varying individual molecule concentrations while keeping the other significant input factors at optimal levels in each condition. At 20% O_2_ levels the predicted specific growth rate is only dependent on input provided by LIF stimulation, and therefore not affected by CHIR concentrations. Under these conditions a concentration range of 600 to 800 U/mL of LIF leads to higher specific growth rates. At 2% O_2_ levels, in addition to LIF, GSK-3 inhibition by CHIR also impacts cell proliferation, and higher specific growth rates are obtained for combinations of LIF at 600–800 U/mL and CHIR at 3–4 µM.
(TIF)

Figure S3 **Cell viability at different oxygen tensions in the presence of LIF and PD, or with LIF plus dual inhibition of MEK/ERK and GSK-3.** Cellular apoptosis and necrosis was further evaluated using flow cytometry following FITC-Annexin V/Propidium Iodide staining for LIF + PD or LIF + CHIR + PD (2i + LIF) conditions at 20% and 2% O_2_. Concentrations used: 1000 U/mL of LIF, 0.4 µM of PD, and 3 µM of CHIR.
(TIF)

Figure S4 **Colony-forming efficiency profiles based on the obtained FC-CD models for 20% and 2% O_2_.** Each graphic represents the variation of the maximum CFE obtained by varying individual molecule concentrations while keeping the other input factors at optimal levels in each condition. At 20% O_2_ levels, colony-forming efficiencies depend on input provided by LIF stimulation, GSK-3 inhibition and MEK/ERK inhibition. Under these conditions a concentration range of 600 to 800 U/mL of LIF and 2–3 µM of CHIR leads to maximal colony-forming efficiencies. MEK/ERK signaling inhibition at higher concentrations (PD = 0.8 µM) in combination with LIF and lacking CHIR, also leads to high colony-forming efficiencies but with slightly lower values when compared to LIF-only conditions. At 2% O_2_ levels, in addition to LIF, GSK-3 inhibition by CHIR also impacts colony-forming efficiencies, and maximum values are obtained for a concentration range of CHIR at 3–5 µM and LIF at 1000 U/mL.
(TIF)

Figure S5 **Factorial design model relating signaling input, provided by LIF and PD, and colony-forming efficiencies of mES cells cultured at 20% O_2_.** Two-factor interaction heat map relating colony-forming efficiency response at 20% O_2_ and signaling input provided by LIF and PD. For LIF: 0 U/mL (−1 level) ≤ [LIF] ≤1000 U/mL (1 level); for PD: 0 µM (−1 level) ≤ [PD] ≤0.8 µM (1 level); for CHIR: [CHIR] = 0 µM (−1 level).
(TIF)

Figure S6 **Hypoxia resulted in reduced levels of key pluripotency genes in mES cells cultured in the absence of GSK-3 inhibition.** Pluripotency markers (*Oct3/4*, *Nanog*, *Rex1* and *Nr0b1*) were evaluated by quantitative PCR following mES cell expansion in KO-DMEM/SR medium supplemented with LIF, LIF plus PD, LIF plus CHIR, or LIF plus the two chemical inhibitors (2i + LIF) at 20% or 2% O_2_ for five consecutive passages. Results are expressed as the average value of two independent experiments performed in duplicate and are relative to gene expression at 20% O_2_ in LIF. The expression levels of the housekeeping gene *Gapdh* were used as internal control.
(TIF)

Figure S7 **Immunofluorescence staining of mES cell colonies maintained in serum-free medium supplemented with LIF and PD at 20% and 2% O_2_.** After expansion, cells were seeded at clonal densities using the indicated conditions and stained for pluripotency markers Oct4 and Nanog. MEK/ERK inhibition is not able to maintain typical colony morphology and homogeneous expression of both pluripotency markers. [LIF] = 1000 U/mL, [PD] = 0.4 µM. Scale bar: 50 µm.
(TIF)

Figure S8 **Histogram plots of phosphorylated and total GSK-3 levels in mES cells maintained at different oxygen tensions and using distinctive signaling input.** Cells were cultured for five consecutive passages using 1000 U/mL of LIF (A), 1000 U/mL of LIF plus 3 µM of CHIR (B), or 1000 U/mL of LIF plus 0.4 µM of PD (C) at 20% and 2% O_2_, and analyzed for both phosphorylated and total GSK-3 levels by flow cytometry. Red, filled histogram − Cells stained for GSK-3α/β or phospho-GSK-3α/β; Blue, open histogram − Cells incubated with control Alexa Fluor 488-conjugated goat-anti rabbit IgG antibody.
(TIF)

Figure S9 **Hypoxia resulted in reduced mES cell proliferation in the absence of GSK-3 inhibition, but CHIR alone is not able to restore typical expansion levels.** Mouse ES cells were cultured in KO-DMEM/SR medium supplemented with the indicated molecules ([LIF] = 1000 U/mL, [CHIR] = 3 µM). Results express the cumulative fold increase in total cell number of five consecutive passages performed in triplicate.
(TIF)

Figure S10 **Immunofluorescence staining of mES cells maintained in serum-free medium supplemented with CHIR at 20% and 2% O_2_.** Cells were expanded for five consecutive passages using CHIR supplementation alone ([CHIR] = 3 µM), and were then stained for pluripotency markers Oct4 and Nanog. GSK-3 inhibition alone is not able to maintain typical cell proliferation levels, colony morphology and homogeneous expression of both pluripotency markers. Scale bar: 50 µm.
(TIF)

Table S1 Coded levels and concentration values of each variable of the two-level face-centered cube experimental design. (C_0_, central point).
(DOC)

Table S2 mES cell specific growth rate (SGR) and colony-forming efficiency (CFE) at 2% and 20% oxygen tensions for each experiment of the two-level face-centered cube design (FC-CD). (C_0_, central point).
(DOC)

Table S3 Results of the analysis of variance (ANOVA) performed to the mES cell specific growth rate (SGR) reduced models.
(DOC)

Table S4 Results of the analysis of variance (ANOVA) performed to the mES cell colony-forming efficiency (CFE) reduced models.
(DOC)

Table S5 Real-time PCR primer sequences used to assess gene expression through relative quantification.
(DOC)

File S1 **Supporting Methods.**
(DOC)
