# Supplementary figures and images for: New Insights into the Mechanisms of Embryonic Stem Cell Self-Renewal under Hypoxia: A Multifactorial Analysis Approach
Source: PLoS One. 2012 Jun 11;7(6):e38963. doi: 10.1371/journal.pone.0038963 (PMC3372480; doi:10.1371/journal.pone.0038963)

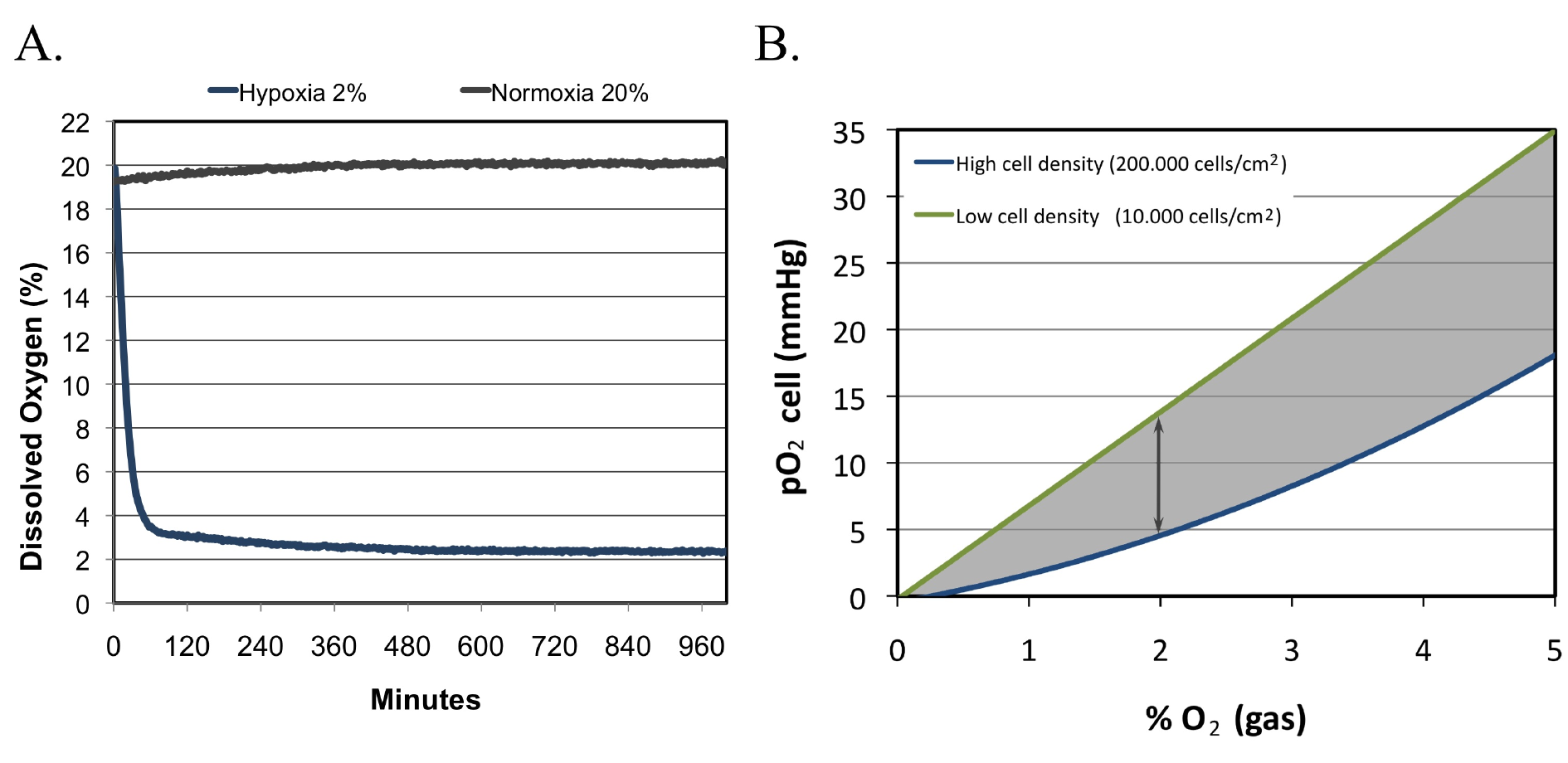

Supplement: Figure S1 — Determination of dissolved oxygen levels in liquid culture medium and estimation of oxygen partial pressure at the cell level (pO2 cell). Measurements were made in 1 mL of KO-DMEM/SR medium and O2 levels were plotted as a function of time (A). The liquid was initially equilibrated at atmospheric oxygen levels, and the plate was then transferred to a humidified incubator at different levels of oxygen in the gaseous phase. Under 20% O2 the dissolved oxygen at the bottom of the culture medium layer was similar to the gas phase tension. As oxygen gas phase levels were reduced (to 2% O2) it was possible to observe a swift reduction in dissolved oxygen within the first hour. The dissolved O2 continued to drop at later times, and eventually reached equilibrium with the gas phase after approximately 5 hours of incubation. Based on the literature, pO2 cell values were obtained for low cell densities (1×10^4^ cells/cm2) and high cell densities (2×10^5^ cells/cm2) and plotted as a function of oxygen levels in the gas phase (B). The corresponding response curves are represented in the graphic for both cell densities. The grey area between both curves represents the expected decrease in pO2 cell levels with increasing cell densities at lower oxygen tensions (up to 5% O2). The grey arrow represents the estimated pO2 cell range for our system at 2% O2. (TIF) [file pone.0038963.s001.tif]

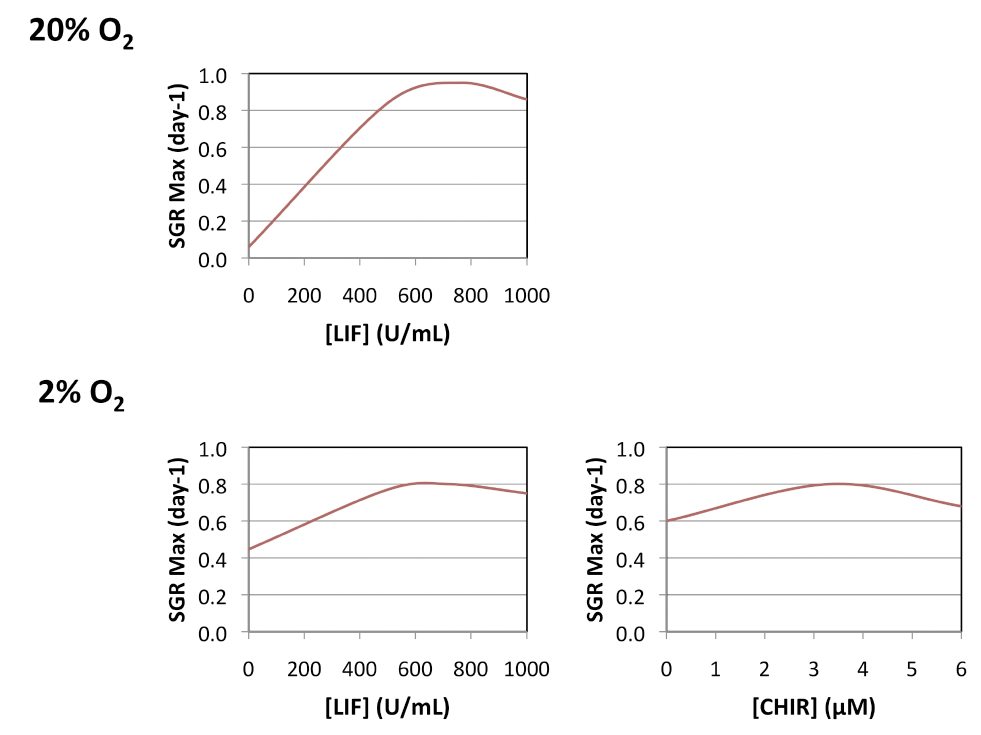

Supplement: Figure S2 — Maximum specific growth rate profiles obtained for 20% and 2% O2 FC-CD models. Each graphic represents the predicted variation of the maximum SGR obtained by varying individual molecule concentrations while keeping the other significant input factors at optimal levels in each condition. At 20% O2 levels the predicted specific growth rate is only dependent on input provided by LIF stimulation, and therefore not affected by CHIR concentrations. Under these conditions a concentration range of 600 to 800 U/mL of LIF leads to higher specific growth rates. At 2% O2 levels, in addition to LIF, GSK-3 inhibition by CHIR also impacts cell proliferation, and higher specific growth rates are obtained for combinations of LIF at 600–800 U/mL and CHIR at 3–4 µM. (TIF) [file pone.0038963.s002.tif]

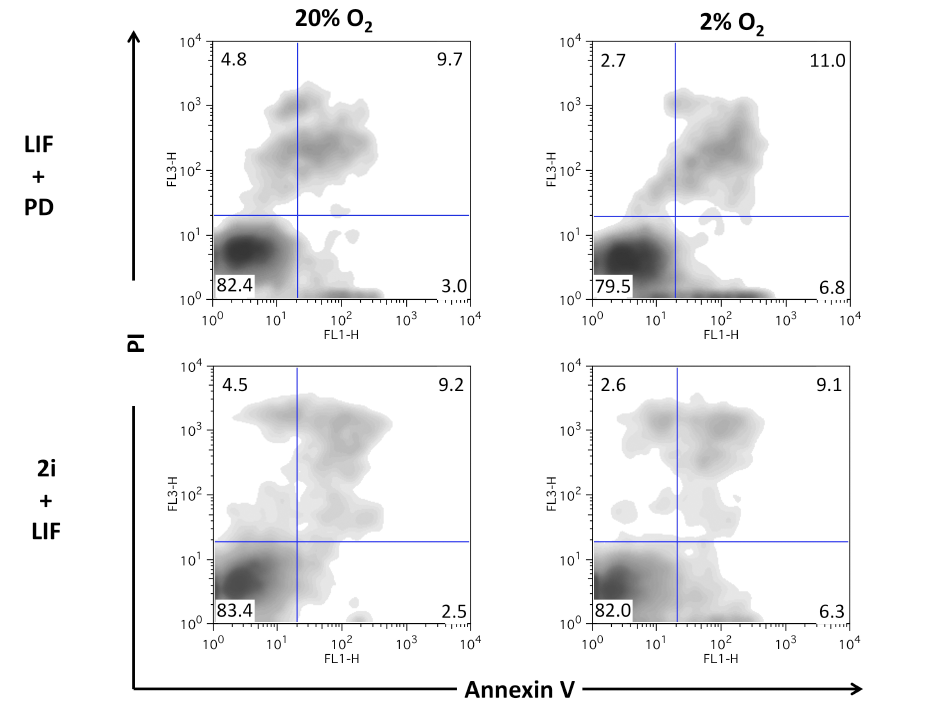

Supplement: Figure S3 — Cell viability at different oxygen tensions in the presence of LIF and PD, or with LIF plus dual inhibition of MEK/ERK and GSK-3. Cellular apoptosis and necrosis was further evaluated using flow cytometry following FITC-Annexin V/Propidium Iodide staining for LIF + PD or LIF + CHIR + PD (2i + LIF) conditions at 20% and 2% O2. Concentrations used: 1000 U/mL of LIF, 0.4 µM of PD, and 3 µM of CHIR. (TIF) [file pone.0038963.s003.tif]

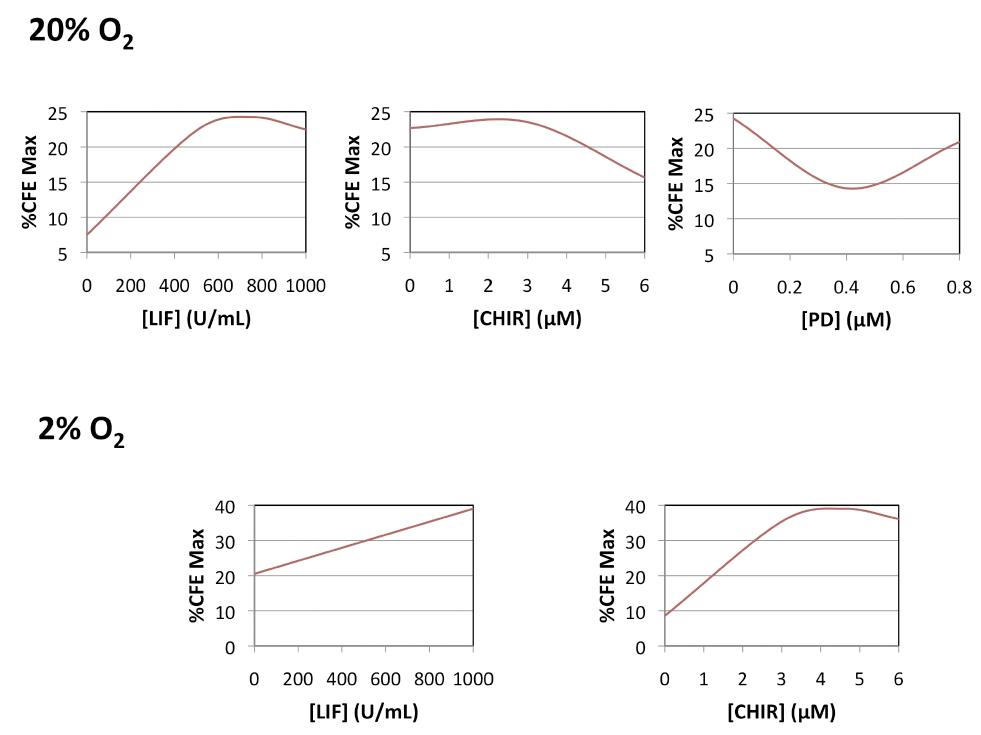

Supplement: Figure S4 — Colony-forming efficiency profiles based on the obtained FC-CD models for 20% and 2% O2. Each graphic represents the variation of the maximum CFE obtained by varying individual molecule concentrations while keeping the other input factors at optimal levels in each condition. At 20% O2 levels, colony-forming efficiencies depend on input provided by LIF stimulation, GSK-3 inhibition and MEK/ERK inhibition. Under these conditions a concentration range of 600 to 800 U/mL of LIF and 2–3 µM of CHIR leads to maximal colony-forming efficiencies. MEK/ERK signaling inhibition at higher concentrations (PD = 0.8 µM) in combination with LIF and lacking CHIR, also leads to high colony-forming efficiencies but with slightly lower values when compared to LIF-only conditions. At 2% O2 levels, in addition to LIF, GSK-3 inhibition by CHIR also impacts colony-forming efficiencies, and maximum values are obtained for a concentration range of CHIR at 3–5 µM and LIF at 1000 U/mL. (TIF) [file pone.0038963.s004.tif]

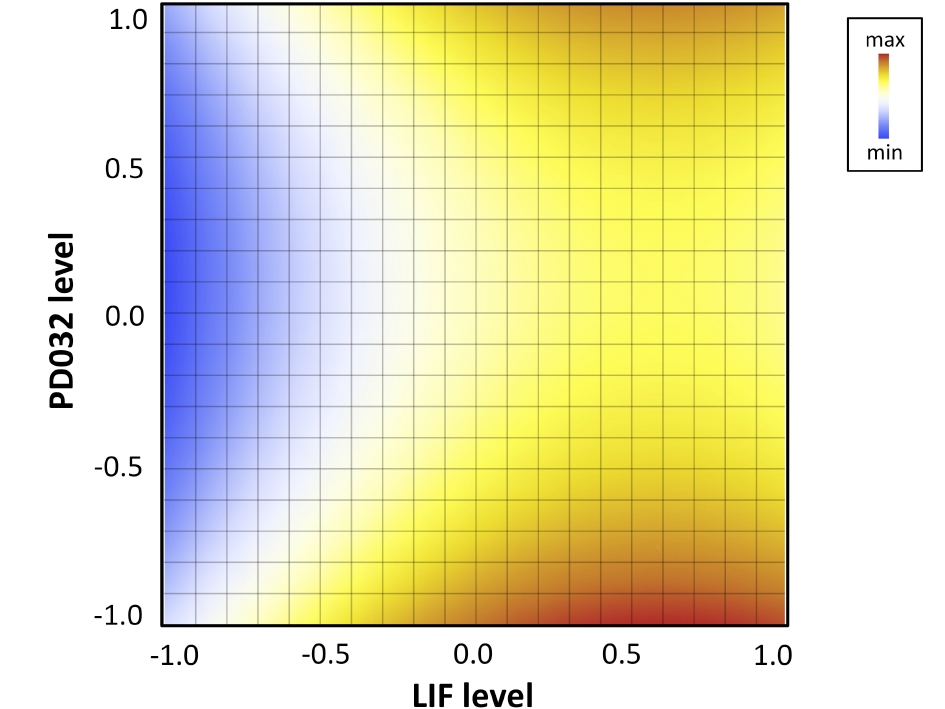

Supplement: Figure S5 — Factorial design model relating signaling input, provided by LIF and PD, and colony-forming efficiencies of mES cells cultured at 20% O2. Two-factor interaction heat map relating colony-forming efficiency response at 20% O2 and signaling input provided by LIF and PD. For LIF: 0 U/mL (−1 level) ≤ [LIF] ≤1000 U/mL (1 level); for PD: 0 µM (−1 level) ≤ [PD] ≤0.8 µM (1 level); for CHIR: [CHIR] = 0 µM (−1 level). (TIF) [file pone.0038963.s005.tif]

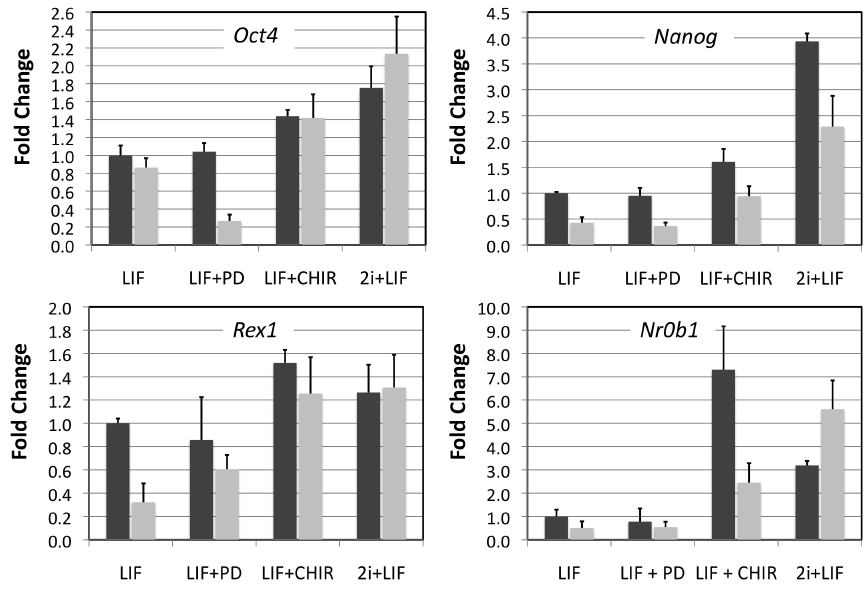

Supplement: Figure S6 — Hypoxia resulted in reduced levels of key pluripotency genes in mES cells cultured in the absence of GSK-3 inhibition. Pluripotency markers (Oct3/4, Nanog, Rex1 and Nr0b1) were evaluated by quantitative PCR following mES cell expansion in KO-DMEM/SR medium supplemented with LIF, LIF plus PD, LIF plus CHIR, or LIF plus the two chemical inhibitors (2i + LIF) at 20% or 2% O2 for five consecutive passages. Results are expressed as the average value of two independent experiments performed in duplicate and are relative to gene expression at 20% O2 in LIF. The expression levels of the housekeeping gene Gapdh were used as internal control. (TIF) [file pone.0038963.s006.tif]

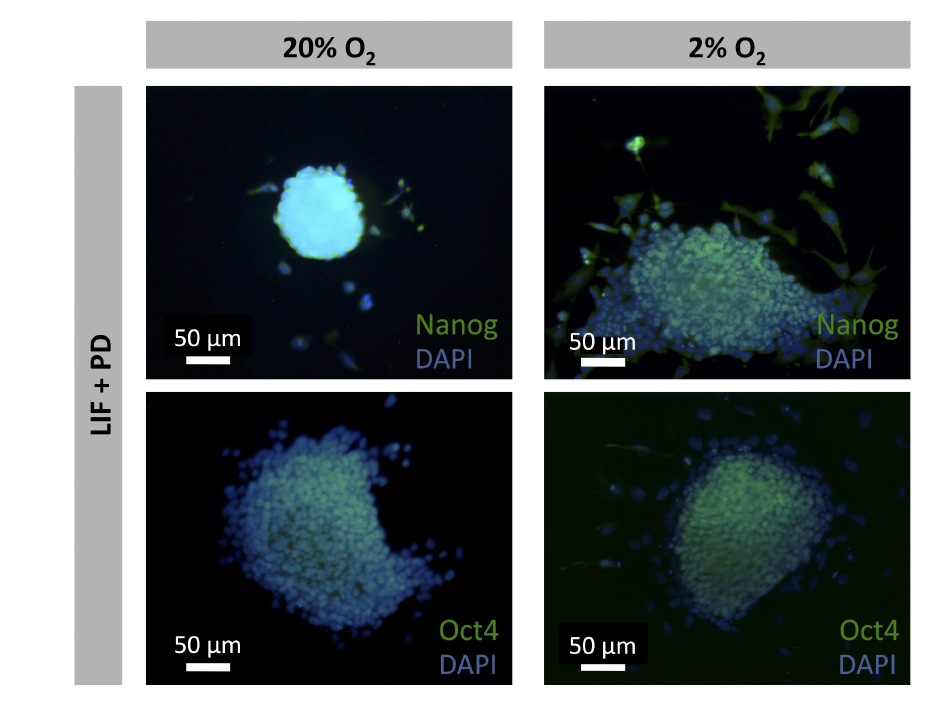

Supplement: Figure S7 — Immunofluorescence staining of mES cell colonies maintained in serum-free medium supplemented with LIF and PD at 20% and 2% O2. After expansion, cells were seeded at clonal densities using the indicated conditions and stained for pluripotency markers Oct4 and Nanog. MEK/ERK inhibition is not able to maintain typical colony morphology and homogeneous expression of both pluripotency markers. [LIF] = 1000 U/mL, [PD] = 0.4 µM. Scale bar: 50 µm. (TIF) [file pone.0038963.s007.tif]

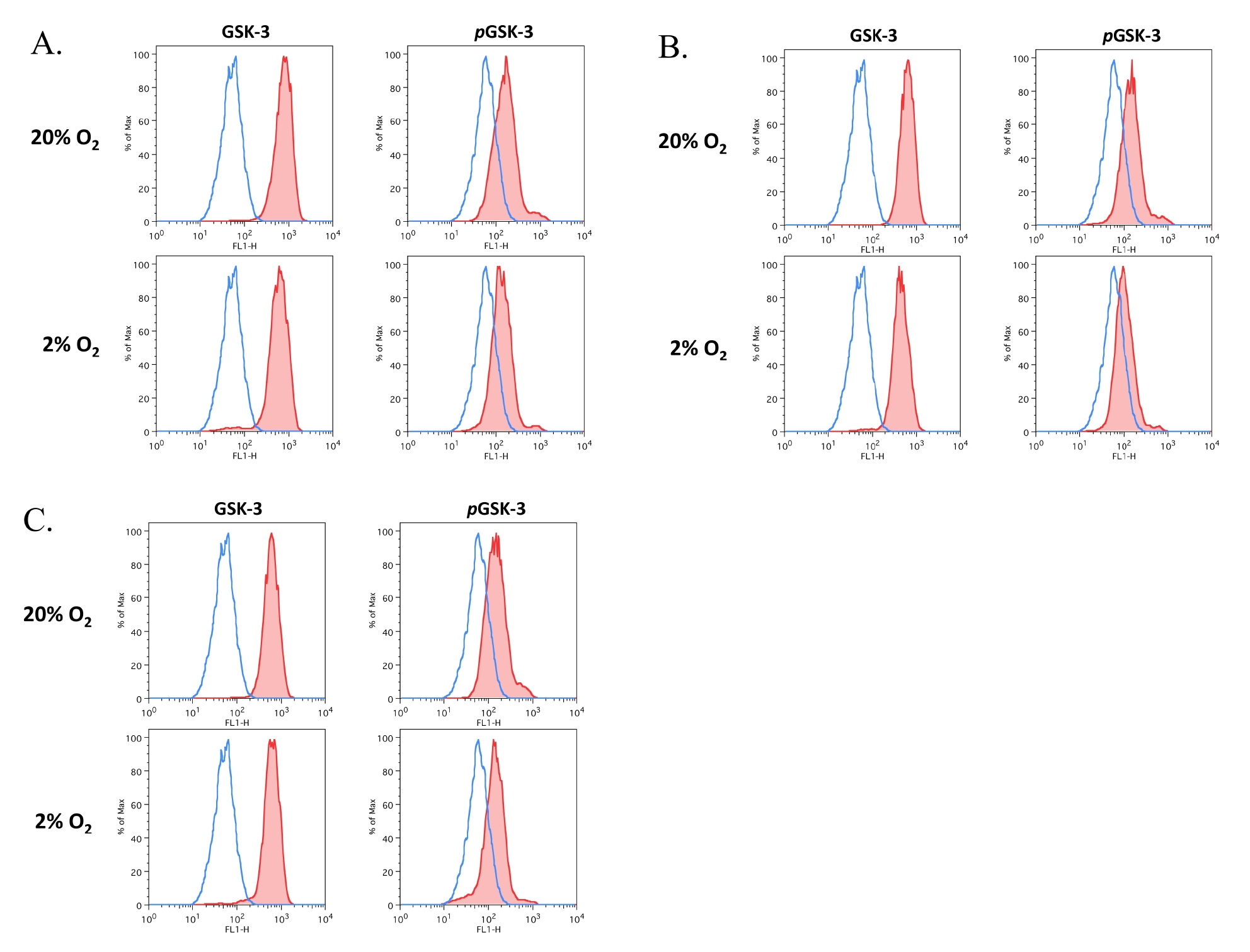

Supplement: Figure S8 — Histogram plots of phosphorylated and total GSK-3 levels in mES cells maintained at different oxygen tensions and using distinctive signaling input. Cells were cultured for five consecutive passages using 1000 U/mL of LIF (A), 1000 U/mL of LIF plus 3 µM of CHIR (B), or 1000 U/mL of LIF plus 0.4 µM of PD (C) at 20% and 2% O2, and analyzed for both phosphorylated and total GSK-3 levels by flow cytometry. Red, filled histogram − Cells stained for GSK-3α/β or phospho-GSK-3α/β; Blue, open histogram − Cells incubated with control Alexa Fluor 488-conjugated goat-anti rabbit IgG antibody. (TIF) [file pone.0038963.s008.tif]

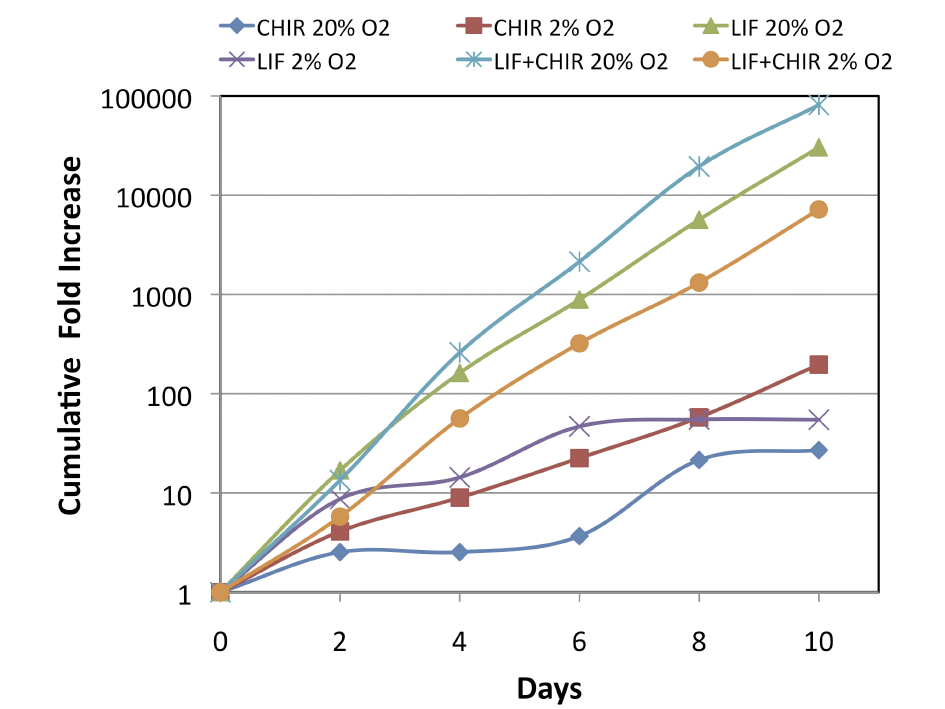

Supplement: Figure S9 — Hypoxia resulted in reduced mES cell proliferation in the absence of GSK-3 inhibition, but CHIR alone is not able to restore typical expansion levels. Mouse ES cells were cultured in KO-DMEM/SR medium supplemented with the indicated molecules ([LIF] = 1000 U/mL, [CHIR] = 3 µM). Results express the cumulative fold increase in total cell number of five consecutive passages performed in triplicate. (TIF) [file pone.0038963.s009.tif]

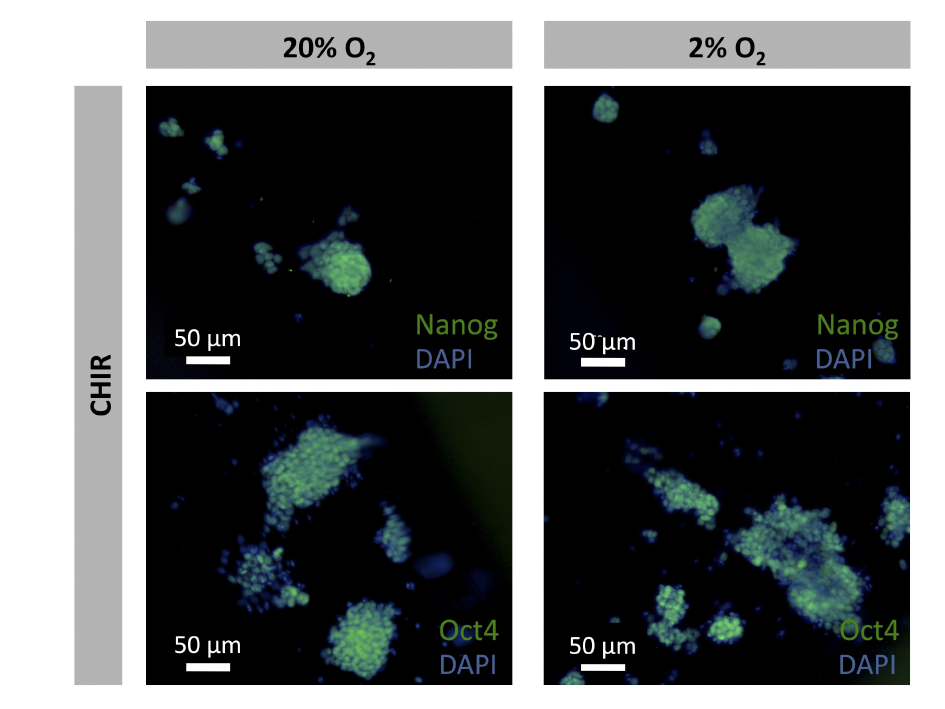

Supplement: Figure S10 — Immunofluorescence staining of mES cells maintained in serum-free medium supplemented with CHIR at 20% and 2% O2. Cells were expanded for five consecutive passages using CHIR supplementation alone ([CHIR] = 3 µM), and were then stained for pluripotency markers Oct4 and Nanog. GSK-3 inhibition alone is not able to maintain typical cell proliferation levels, colony morphology and homogeneous expression of both pluripotency markers. Scale bar: 50 µm. (TIF) [file pone.0038963.s010.tif]
