# Supplementary material for: New Insights into the Mechanisms of Embryonic Stem Cell Self-Renewal under Hypoxia: A Multifactorial Analysis Approach
Source: PLoS One. 2012 Jun 11;7(6):e38963. doi: 10.1371/journal.pone.0038963 (PMC3372480; doi:10.1371/journal.pone.0038963)
Supplement: Table S1 — Coded levels and concentration values of each variable of the two-level face-centered cube experimental design. (C0, central point). (DOC) [file pone.0038963.s011.doc]

**Supporting Table 1:**

**Table S1.** Coded levels and concentration values of each variable of the two-level face-centered cube experimental design. (C_0_, central point).

| **Experimental Run** | **Coded Levels** | | | **Concentration Used** | | |
| --- | --- | --- | --- | --- | --- | --- |
| **#** | **LIF** | **CHIR** | **PD** | **LIF (U/mL)** | **CHIR (µM)** | **PD (µM)** |
| 1 | -1 | -1 | -1 | 0 | 0 | 0 |
| 2 | -1 | -1 | 1 | 0 | 0 | 0.8 |
| 3 | -1 | 1 | -1 | 0 | 6 | 0 |
| 4 | -1 | 1 | 1 | 0 | 6 | 0.8 |
| 5 | 1 | -1 | -1 | 1000 | 0 | 0 |
| 6 | 1 | -1 | 1 | 1000 | 0 | 0.8 |
| 7 | 1 | 1 | -1 | 1000 | 6 | 0 |
| 8 | 1 | 1 | 1 | 1000 | 6 | 0.8 |
| 9 | -1 | 0 | 0 | 0 | 3 | 0.4 |
| 10 | 1 | 0 | 0 | 1000 | 3 | 0.4 |
| 11 | 0 | -1 | 0 | 500 | 0 | 0.4 |
| 12 | 0 | 1 | 0 | 500 | 6 | 0.4 |
| 13 | 0 | 0 | -1 | 500 | 3 | 0 |
| 14 | 0 | 0 | 1 | 500 | 3 | 0.8 |
| 15 (C_0_) | 0 | 0 | 0 | 500 | 3 | 0.4 |
| 16 (C_0_) | 0 | 0 | 0 | 500 | 3 | 0.4 |
| 17 (C_0_) | 0 | 0 | 0 | 500 | 3 | 0.4 |
| 18 (C_0_) | 0 | 0 | 0 | 500 | 3 | 0.4 |
