# Supplementary material for: New Insights into the Mechanisms of Embryonic Stem Cell Self-Renewal under Hypoxia: A Multifactorial Analysis Approach
Source: PLoS One. 2012 Jun 11;7(6):e38963. doi: 10.1371/journal.pone.0038963 (PMC3372480; doi:10.1371/journal.pone.0038963)
Supplement: Table S2 — mES cell specific growth rate (SGR) and colony-forming efficiency (CFE) at 2% and 20% oxygen tensions for each experiment of the two-level face-centered cube design (FC-CD). (C0, central point). (DOC) [file pone.0038963.s012.doc]

**Supporting Table 2:**

**Table S2.** mES cell specific growth rate (SGR) and colony-forming efficiency (CFE) at 2% and 20% oxygen tensions for each experiment of the two-level face-centered cube design (FC-CD). (C_0_, central point).

| **Experimental Run** | **20% O_2_** | | **2% O_2_** | |
| --- | --- | --- | --- | --- |
| **#** | **SGR (Day^-1^)** | **CFE (%)** | **SGR (Day^-1^)** | **CFE (%)** |
| 1 | 0.00 | 0.0 | 0.00 | 0.0 |
| 2 | 0.00 | 0.0 | 0.00 | 0.5 |
| 3 | 0.00 | 0.0 | 0.27 | 0.8 |
| 4 | 0.15 | 0.0 | 0.43 | 4.5 |
| 5 | 0.95 | 18.4 | 0.55 | 8.8 |
| 6 | 0.98 | 20.7 | 0.58 | 10.3 |
| 7 | 0.89 | 15.3 | 0.60 | 39.8 |
| 8 | 0.66 | 6.3 | 0.57 | 37.5 |
| 9 | 0.19 | 3.1 | 0.67 | 17.3 |
| 10 | 0.86 | 11.1 | 0.75 | 19.3 |
| 11 | 0.97 | 14.1 | 0.72 | 10.5 |
| 12 | 0.85 | 6.3 | 0.72 | 18.5 |
| 13 | 0.97 | 27.8 | 0.79 | 17.5 |
| 14 | 0.77 | 18.1 | 0.75 | 22.8 |
| 15 (C_0_) | 0.77 | 10.8 | 0.73 | 36.0 |
| 16 (C_0_) | 0.73 | 10.9 | 0.62 | 34.5 |
| 17 (C_0_) | 0.89 | 7.1 | 0.72 | 34.0 |
| 18 (C_0_) | 0.84 | 8.8 | 0.68 | 27.8 |
