# Supplementary material for: New Insights into the Mechanisms of Embryonic Stem Cell Self-Renewal under Hypoxia: A Multifactorial Analysis Approach
Source: PLoS One. 2012 Jun 11;7(6):e38963. doi: 10.1371/journal.pone.0038963 (PMC3372480; doi:10.1371/journal.pone.0038963)
Supplement: Table S3 — Results of the analysis of variance (ANOVA) performed to the mES cell specific growth rate (SGR) reduced models. (DOC) [file pone.0038963.s013.doc]

**Supporting Table 3:**

**Table S3.** Results of the analysis of variance (ANOVA) performed to the mES cell specific growth rate (SGR) reduced models.

| **Oxygen tension** | **Source** | | | **SS** | **df** | **MS** | **F-value** | **F-critical** | **ρ-value** |
| --- | --- | --- | --- | --- | --- | --- | --- | --- | --- |
|  |  |  |  |  |  |  |  |  |  |
| **20% O_2_** | SGR (Day^-1^) | Regression | | 2.25 | 2 | 1.13 | 109.67 | 9.55 | 0.002 |
|  |  | Residual | | 0.15 | 15 | 0.01 |  |  |  |
|  |  |  | Lack of Fit (LOF) | 0.14 | 12 | 0.01 | 2.24 | 8.74 | 0.276 |
|  |  |  | Pure error (PE) | 0.02 | 3 | 0.01 |  |  |  |
|  |  | Total | | 2.41 | 17 |  |  |  |  |
| **2% O_2_** | SGR (Day^-1^) | Regression | | 0.83 | 5 | 0.17 | 11.18 | 9.01 | 0.037 |
|  |  | Residual | | 0.18 | 12 | 0.01 |  |  |  |
|  |  |  | Lack of Fit (LOF) | 0.17 | 9 | 0.02 | 7.32 | 8.81 | 0.064 |
|  |  |  | Pure error (PE) | 0.01 | 3 | 0.00 |  |  |  |
|  |  | Total | | 1.01 | 17 |  |  |  |  |
