# Supplementary material for: New Insights into the Mechanisms of Embryonic Stem Cell Self-Renewal under Hypoxia: A Multifactorial Analysis Approach
Source: PLoS One. 2012 Jun 11;7(6):e38963. doi: 10.1371/journal.pone.0038963 (PMC3372480; doi:10.1371/journal.pone.0038963)
Supplement: Table S4 — Results of the analysis of variance (ANOVA) performed to the mES cell colony-forming efficiency (CFE) reduced models. (DOC) [file pone.0038963.s014.doc]

**Supporting Table 4:**

**Table S4. Results of the analysis of variance (ANOVA) performed to the mES cell colony-forming efficiency (CFE) reduced models.**

| **Oxygen tension** | **Source** | | | **SS** | **df** | **MS** | **F-value** | **F-critical** | **ρ-value** |
| --- | --- | --- | --- | --- | --- | --- | --- | --- | --- |
| **20% O_2_** | CFE (%) | Regression | | 928.10 | 6 | 154.68 | 8.97 | 8.94 | 0.049 |
|  |  | Residual | | 172.40 | 10 | 17.24 |  |  |  |
|  |  |  | Lack of Fit (LOF) | 162.72 | 7 | 23.25 | 7.21 | 8.89 | 0.066 |
|  |  |  | Pure error (PE) | 9.67 | 3 | 3.22 |  |  |  |
|  |  | Total | | 1100.50 | 16 |  |  |  |  |
| **2% O_2_** | CFE (%) | Regression | | 2471.51 | 4 | 617.88 | 12.32 | 9.12 | 0.033 |
|  |  | Residual | | 652.02 | 13 | 50.16 |  |  |  |
|  |  |  | Lack of Fit (LOF) | 612.22 | 10 | 61.22 | 4.62 | 8.79 | 0.117 |
|  |  |  | Pure error (PE) | 39.80 | 3 | 13.27 |  |  |  |
|  |  | Total | | 3123.53 | 17 |  |  |  |  |
