# Supplementary material for: New Insights into the Mechanisms of Embryonic Stem Cell Self-Renewal under Hypoxia: A Multifactorial Analysis Approach
Source: PLoS One. 2012 Jun 11;7(6):e38963. doi: 10.1371/journal.pone.0038963 (PMC3372480; doi:10.1371/journal.pone.0038963)
Supplement: Table S5 — Real-time PCR primer sequences used to assess gene expression through relative quantification. (DOC) [file pone.0038963.s015.doc]

**Supporting Table 5:**

**Table S5.** Real-time PCR primer sequences used to assess gene expression through relative quantification.

| ***Gene Name*** | ***Primer Sequences*** |
| --- | --- |
| ***Oct4*** | Fwd: 5'- gcc ttg cag ctc agc ctt aa -3'  Rev: 5'- ctc att gtt gtc ggc ttc ctc -3' |
| ***Nanog*** | Fwd: 5'- atg cct gca gtt ttt cat cc -3'  Rev: 5'- gag gca ggt ctt cag agg aa -3' |
| ***Rex1*** | Fwd: 5'- aga ttt cca ctg tgg ctc tgg gta -3'  Rev: 5'- ctt cca ggg ggc act gat ccg -3' |
| ***Nr0b1*** | Fwd: 5'- acg cgt ctc agg aag agc ga -3'  Rev: 5'- gtg ctc cga atc gtg ccc tc -3' |
| ***Gapdh*** | Fw: 5'-gca cag tca agg ccg aga at-3'  Re: 5'-gcc ttc tcc atg gtg gtg aa-3' |
