# Supplementary material for: New Insights into the Mechanisms of Embryonic Stem Cell Self-Renewal under Hypoxia: A Multifactorial Analysis Approach
Source: PLoS One. 2012 Jun 11;7(6):e38963. doi: 10.1371/journal.pone.0038963 (PMC3372480; doi:10.1371/journal.pone.0038963)
Supplement: File S1 — Supporting Methods. (DOC) [file pone.0038963.s016.doc]

**Supporting Methods**

**“New Insights into the Mechanisms of Embryonic Stem Cell Self-Renewal under Hypoxia: a Multifactorial Analysis Approach”**

Hélder S.C. Barbosa, Tiago G. Fernandes, Tiago P. Dias,

Maria Margarida Diogo, Joaquim M.S. Cabral

Department of Bioengineering, and Institute for Biotechnology and Bioengineering, Centre for Biological and Chemical Engineering, Instituto Superior Técnico, Technical University of Lisbon, Lisboa, Portugal.

**Supporting Methods**

**Cell line**

In this study the 46C mouse ES cell line (a kind gift from professor Austin Smith, University of Cambridge, UK) was used as model system. The 46C cell line contains a green fluorescent protein (GFP) gene knock-in at the Sox1 locus (neuroepithelial marker gene) and can be induced to undergo neural commitment in serum-free conditions . 46C mES cells were maintained in liquid nitrogen prior to use.

**Determination of dissolved oxygen levels in liquid culture medium and estimation of oxygen partial pressure at the cell level (pO_2_ cell)**

To examine the response of dissolved oxygen levels in the liquid medium under simulated culture conditions, a 24-channel SDR SensorDish® Reader (PreSens) for on-line monitoring was used to measure O_2_ tension at the bottom of a liquid layer in multi-well culture plates with integrated optical-chemical oxygen sensors. The liquid was initially equilibrated at atmospheric oxygen levels, and the plate was then transferred to a humidified incubator either at 20% or 2% O_2_ gas phase. The dissolved O_2_ levels were measured as a function of time (Figure S1.A). Estimation of oxygen partial pressure at the cell level (pO_2_ cell) was made for our system based on previously published data by Powers and coworkers . We have fitted these literature values to a polynomial regression curve (first order for low cell densities, r^2^ = 0.999; second order for high cell densities, r^2^ = 0.990), and plotted as a function of oxygen levels in the gas phase (Figure S1.B). The corresponding response curves are represented in the graphic for both cell densities. Although our highest cell density obtained at 2% O_2_ (~100.000 cels/cm^2^) was about half of the high-density value used by Powers et al. in their calculations (200.000 cells/cm^2^), we cannot exclude that pO_2_ cell in our system was also lower than the levels of dissolved oxygen (indicated by a grey arrow in Figure S1.B).

**Cell division analysis**

Cell division analysis was performed using the PKH26 red fluorescent cell linker kit for cell membrane labeling (Sigma). PKH26 molecules are stably integrated in the cell membrane and, when the cell divides, are equally distributed between the daughter cells. Consequently, each daughter cell has half of the fluorescence of their precursors, allowing the determination of the number of cell divisions.

To accurately track mES cell proliferation kinetics in culture, we limited the cell division analysis to one cell passage (2 days). Therefore, after expansion for five consecutive passages (10 days) under designated conditions, cells were collected and stained with PKH26 according to the manufacturer’s instructions. 5 x 10^5^ cells were used for flow cytometry analysis (“Day 0”) and the remaining cells were plated as described. After 2 days in culture (one additional passage under the same designated conditions), cells were collected and analyzed by flow cytometry (FACSCalibur flow cytometer; Becton Dickinson) using the CellQuest software and the Wizard of ModFit software. The software applies an algorithm that assumes cell duplication as cells proliferate through each daughter generation. The proliferation index is automatically calculated based on the number of cells in all generations divided by the computed number of original cells present at the start of the experiment. It is a measurement of the increase in cell number over time. The percentage of cells in the parental generation represents the fraction of cells that have not proliferated during the course of the experiment, and was also calculated using the Wizard of ModFit Software.

**Intracellular staining for immunofluorescence microscopy**

Immunostaining was used to assess the expression and localization of Oct4 and Nanog when mES cells were cultured under different conditions. Colonies were fixed with 4% (w/v) paraformaldehyde solution (Sigma) in PBS. Then, cells were permeabilized and blocked with 10% (v/v) normal goat serum (NGS; Sigma)/0.1% (v/v) Triton-X (Sigma) solution for 30-60 min at room temperature. After three washing cycles with PBS, the primary antibody was added to the cells and overnight incubation was performed. The primary antibodies used were a mouse monoclonal anti-Oct3/4 and a rabbit polyclonal anti-Nanog (both from Millipore), 1:500 (v/v) diluted in blocking solution (10% NGS/0.1% Triton-X in PBS). After removing the excess of primary antibody, incubation for one hour with the secondary antibody (Alexa Fluor 488-conjugated goat-anti mouse or goat-anti rabbit IgG; Molecular Probes), 1:1000 diluted in blocking solution, was performed. After removing the excess of secondary antibody, DAPI (Sigma) staining was executed for 3 minutes. Cells were thoroughly washed with PBS and photos acquired with a fluorescence optical microscope (Leica DMI 3000B, Germany) equipped with a digital camera (Nikon DXM 1200 F).

**Real-time PCR**

Real-time PCR was used to quantify mES cell pluripotency markers. Total RNA was isolated from each sample using the High Pure RNA Isolation Kit (Roche) including DNAse I treatment according to the manufacturer’s instructions, and first-strand cDNA was synthesized using the Transcriptor First-Strand cDNA Synthesis Kit (Roche) with anchored-oligo(dt)18 primers and 1µg of RNA.

Real-time PCR was performed in a Roche Light Cycler detection system using a final reaction volume of 20 µL containing 2.0 mM MgCl_2_ solution, 0.1 µM of each primer, 2 µL of sample and a SYBR green I mixture. The denaturation temperature used was 95ºC, the annealing temperature was 60ºC and the elongation temperature was 70ºC. Each step (denaturation, annealing and elongation) ran for 10 seconds and forty cycles were performed after an initial full denaturation step at 95ºC for 10 minutes. The primer sequences used are shown in Table S5.

Threshold cycle (Ct) values were calculated using LightCycler software 3.4 (Roche) and the Fit Points method. *Gapdh* was used as control housekeeping gene. Relative quantification of gene expression was performed using the delta-delta threshold cycle method (ΔΔCt).

**Apoptosis analysis**

Cellular apoptosis was evaluated using the FITC-Annexin V Apoptosis Detection Kit I (BD Pharmigen). For that purpose, cells were collected, washed twice with PBS, and resuspended in 1x Annexin V binding buffer at a concentration of 10^6^ cells/mL. Then, 10^5^ cells were placed into a FACS tube, marked with FITC-Annexin V and Propidium Iodide staining solution, and incubated for 15 min at room temperature in the dark. After incubation, 400 µL of 1x Annexin V binding buffer were added and the samples were analyzed within an hour. Proper controls were prepared with cells, cells marked only with FITC-Annexin V, and cells marked only with Propidium Iodide (PI).

Samples were run in a FACSCalibur Flow Cytometer (Becton Dickinson) and analyzed using the CellQuest Software. Cell debris were excluded from the analysis using forward scatter (cell size) and side scatter (cell complexity) criteria. A minimum of 10000 events was collected for each sample.

**Intracellular immunofluorescence staining for flow cytometry**

GSK-3 phosphorylation was analyzed using intracellular immunofluorescence staining and flow cytometry. For that, cells were fixed with 2% (w/v) paraformaldehyde solution (Sigma) in PBS. Then, cells were permeabilized with 1% (w/v) saponin solution (Sigma) in PBS, followed by 15 min incubation with 3% (v/v) normal goat serum solution (NGS; Sigma) in PBS. After the washing steps, the primary antibodies were added, and cells incubated for 2 hours. The primary antibodies used were a polyclonal rabbit anti-human/mouse/rat GSK-3α/β antibody (R&D Systems), diluted in blocking solution at a concentration of 10 μg/mL, and a monoclonal rabbit anti-phospho-GSK-3α/β (S21/S9) antibody (R&D Systems), diluted in blocking solution at a concentration of 25 μg/mL. After removing the excess of primary antibody, cells were incubated for one hour with the secondary antibody (Alexa Fluor 488-conjugated goat-anti rabbit IgG; Molecular Probes, 1:1000 dilution in blocking solution).

The acquisition of the samples was performed in a FACSCalibur Flow Cytometer (Becton Dickinson) and analyzed using the CellQuest Software. Cell debris were excluded from the analysis using forward scatter (cell size) and side scatter (cell complexity) criteria. A minimum of 10000 events was collected for each sample.

**Supporting Bibliography**

1. Ying QL, Stavridis M, Griffiths D, Li M, Smith A (2003) Conversion of embryonic stem cells into neuroectodermal precursors in adherent monoculture. Nat Biotechnol 21: 183-186.

2. Diogo MM, Henrique D, Cabral JM (2008) Optimization and integration of expansion and neural commitment of mouse embryonic stem cells. Biotechnol Appl Biochem 49: 105-112.

3. Powers DE, Millman JR, Huang RB, Colton CK (2008) Effects of oxygen on mouse embryonic stem cell growth, phenotype retention, and cellular energetics. Biotechnol Bioeng 101: 241-254.
